# Dipeptide species regulate p38MAPK–Smad3 signalling to maintain chronic myelogenous leukaemia stem cells

**DOI:** 10.1038/ncomms9039

**Published:** 2015-08-20

**Authors:** Kazuhito Naka, Yoshie Jomen, Kaori Ishihara, Junil Kim, Takahiro Ishimoto, Eun-Jin Bae, Robert P. Mohney, Steven M. Stirdivant, Hiroko Oshima, Masanobu Oshima, Dong-Wook Kim, Hiromitsu Nakauchi, Yoshihiro Takihara, Yukio Kato, Akira Ooshima, Seong-Jin Kim

**Affiliations:** 1 Exploratory Project on Cancer Stem Cells, Cancer Research Institute, Kanazawa University, Kakuma-machi, Kanazawa, Ishikawa 920-1192, Japan; 2 Department of Stem Cell Biology, Research Institute for Radiation Biology and Medicine, Hiroshima University, 1-2-3 Kasumi, Minami-ku, Hiroshima 734-8553, Japan; 3 CHA Cancer Institute and Department of Biomedical Science, CHA University, CHA Bio Complex, 335 Pangyo-ro, Bundang-ku, Seongnam, Kyunggi-do 463-400, Republic of Korea; 4 Faculty of Pharmacy, Institute of Medical, Pharmaceutical and Health Sciences, Kanazawa University, Kakuma-machi, Kanazawa, Ishikawa 920-1192, Japan; 5 Metabolon, Inc., 617 Davis Drive Suite 400, Durham, North Carolina 27713, USA; 6 Division of Genetics, Cancer Research Institute, Kanazawa University, Kakuma-machi, Kanazawa, Ishikawa 920-1192, Japan; 7 Department of Hematology, Seoul St Mary's Hospital, Cancer Research Institute, The Catholic University of Korea, 222 Banpo-daero, Seocho-gu, Seoul 137-701, Republic of Korea; 8 Division of Stem Cell Therapy, Center for Stem Cell Biology and Regeneration Medicine, Institute of Medical Science, The University of Tokyo, 4-6-1 Shiroganedai, Minato-ku, Tokyo 108-8639, Japan; 9 Institute for Stem Cell Biology and Regenerative Medicine, Stanford University School of Medicine, 265 Campus Drive, Stanford, California 94305, USA

## Abstract

Understanding the specific survival of the rare chronic myelogenous leukaemia (CML) stem cell population could provide a target for therapeutics aimed at eradicating these cells. However, little is known about how survival signalling is regulated in CML stem cells. In this study, we survey global metabolic differences between murine normal haematopoietic stem cells (HSCs) and CML stem cells using metabolomics techniques. Strikingly, we show that CML stem cells accumulate significantly higher levels of certain dipeptide species than normal HSCs. Once internalized, these dipeptide species activate amino-acid signalling via a pathway involving p38MAPK and the stemness transcription factor Smad3, which promotes CML stem cell maintenance. Importantly, pharmacological inhibition of dipeptide uptake inhibits CML stem cell activity *in vivo*. Our results demonstrate that dipeptide species support CML stem cell maintenance by activating p38MAPK–Smad3 signalling *in vivo*, and thus point towards a potential therapeutic target for CML treatment.

## Abstract (web summary)

Chronic myelogenous leukaemia contains a stem cell fraction and targeting this population of cells is an attractive therapeutic strategy. Here, the authors demonstrate that the stem cells take up dipeptides and that inhibiting the dipeptide transporter could reduce the number of these stem cells in mice.

## Graphical abstract

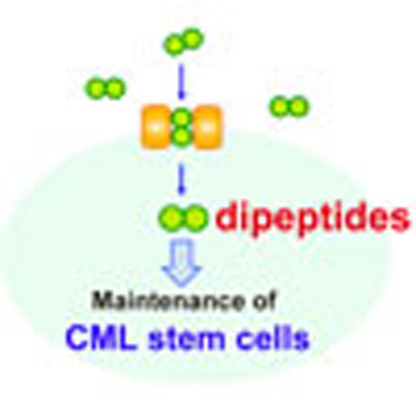

Chronic myelogenous leukaemia (CML) arises when the *BCR-ABL1* oncogene is generated in haematopoietic stem cells (HSCs)^1^. Although tyrosine kinase inhibitors (TKIs), such as the first-generation TKI imatinib mesylate (IM) and the second-generation TKIs dasatinib and nilotinib, have markedly improved the prognosis of CML patients, a cure remains elusive^2^,^3^,^4^,^5^. CML stem cells, which are the cellular source of the vast majority of differentiated CML cells, are reportedly responsible for the recurrence of CML disease following TKI therapy^1^,^6^,^7^. Thus, to completely eradicate quiescent CML stem cells and CML disease, TKIs may have to be coupled with novel therapeutics targetting alternative molecular pathways.

A nutrient supply specifically required for CML stem cell maintenance could provide a candidate target for a novel therapy capable of eradicating CML stem cells. However, to reduce the harmful side effects of such molecular targetting on normal haematopoiesis, it is essential to understand the altered mechanisms that distinguish CML stem cells from normal HSCs. To pinpoint CML-associated nutrient signalling, we carried out a global metabolic comparison of normal HSCs with the corresponding stages of CML stem cells in tetracycline (tet)-inducible CML-affected mice^8^,^9^,^10^. Our approach allowed us to use doxycycline (DOX) withdrawal to synchronize the induction of CML disease in these mice via HSC-specific activation of the tTA (tetracycline-controlled transactivator) protein, and to obtain the most primitive long-term (LT)-CML stem cells from the bone marrow (BM) of animals developing CML. This strategy of metabolic analysis in a well-characterized CML model has uncovered a nutrient signalling pathway that is critical for the *in vivo* maintenance of CML stem cells but not normal HSCs.

In mammals, the uptake of small peptides by the Slc15A family of oligo/dipeptide transporters provides an effective and energy-saving intracellular source of amino acids^11^,^12^,^13^. These transporters are encoded by the *Slc15A1* (previously designated *Pept1*), *Slc15A2* (*Pept2*), *Slc15A4* (*Pht1*) and *Slc15A3* (*Pht2*) genes. Oligo-/dipeptide uptake dependent on Slc15A1/2 has been well studied in renal and intestinal epithelial cells^11^,^12^,^13^, but the functions of these transporters in haematopoietic cells are obscure. We propose that Slc15A2 dipeptide transporter activity sustains CML stem cell maintenance by guaranteeing an alternative nutrient supply. Most importantly, this survival mechanism apparently does not operate routinely in normal HSCs.

CML stem cell maintenance is also influenced by the TGF-β pathway, which can either decrease or increase CML stem cell numbers *in vivo* depending on the cellular context^14^,^15^. Because Smad3, a downstream effector of TGF-β signalling, is a ‘master regulator' of cell fate^16^, it has been of great interest to determine whether Smad3 promotes the maintenance of ‘stemness' *in vivo*, including CML cell stemness. In our study, we provide evidence that post-translational modification of Smad3 by non-canonical phosphorylation at Ser208 is crucial for CML stem cell activity *in vivo*. Intriguingly, we also demonstrate that this non-canonical Smad3 phosphorylation is mediated by dipeptide-triggered activation of p38MAPK.

Our results demonstrate that dipeptide species support a nutrient signalling mechanism that is required for CML stem cell activity *in vivo*. This novel mechanism has two linked features: (1) primitive CML stem cells take up dipeptide species through Slc15A2 transporter activity, and (2) these internalized dipeptides regulate nutrient signalling pathway(s) through p38MAPK-mediated Smad3–Ser208 phosphorylation. Because this mechanism does not appear to function in normal HSCs, it may be possible to eliminate vulnerable CML stem cells by therapeutic targetting of this crucial nutrient signalling pathway, offering new hope for reducing CML recurrence in patients.

## Results

### CML stem cells accumulate several dipeptide species

**Figure 1:**
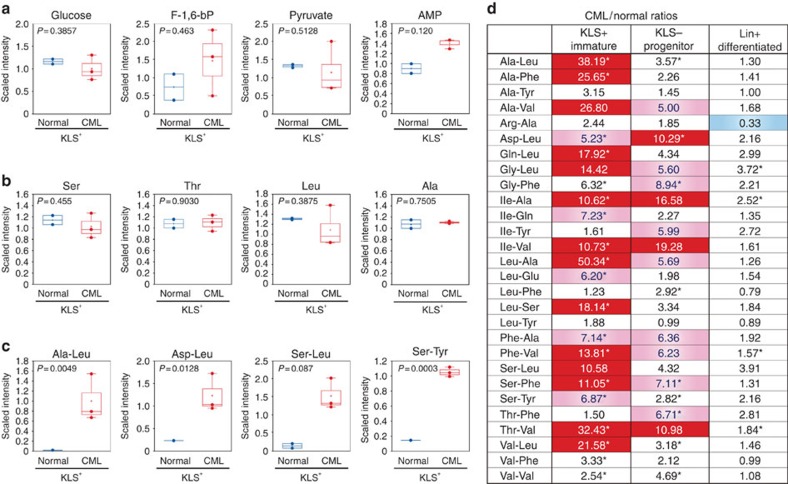
CML stem cells show accumulations of dipeptides not found in normal HSCs. Metabolomic analyses of KLS^+^, KLS^−^ and Lin^+^ cells from CML-affected (*Tal1-tTA*^*+*^*TRE-BCR-ABL1*^*+*^) mice (male, *n*=6; female, *n*=6; three experiments) and normal healthy (*Tal1-tTA*^*+*^) littermates (male, *n*=6; female, *n*=6; two experiments) at 5 weeks post DOX withdrawal. Data are representative of three independent trials. (**a**–**c**) Amounts of (**a**) metabolites related to glycolysis, (**b**) amino acids and (**c**) dipeptides in KLS^+^ cells were plotted in whisker boxes. Cross, mean value; horizontal line across box, median value; error bars, maximum and minimum of distribution; dot, extreme data point. *P* value indicates the statistical significance among normal KLS^+^ versus CML-KLS^+^ as measured by Welch's *t*-test. (**d**) Ratios of the indicated dipeptide levels in CML versus normal haematopoietic cells of the indicated subsets. Red-shaded values indicate an increase in expression in CML cells of >10-fold; light red, >5-fold; light blue, <0.5-fold (*P<0.05; normal versus CML; Welch's *t*-test).

To identify nutrient signalling differences between normal HSCs and CML stem cells, we carried out a global metabolic comparison of cells isolated from control and tet-inducible CML-affected mice. To obtain the latter animals, we crossed *Tal1-tTA* mice with *TRE-BCR-ABL1* transgenic mice (FVB/N background) to generate *Tal1-tTA* × *TRE-BCR-ABL1* double-transgenic progeny^8^,^9^,^10^,^17^,^18^. When these progeny are subjected to DOX withdrawal, synchronous induction of CML disease occurs with the generation of CML stem cells. From healthy control (*Tal1-tTA*^*+*^) and CML-affected (*Tal1-tTA*^*+*^*TRE-BCR-ABL1*^*+*^) littermates, we isolated the following cell subsets: the immature KLS^+^ (cKit^+^Lineage^−^Sca-1^+^) population, which includes normal HSCs and CML stem cells (also known as leukaemia-initiating cells (LICs)), the committed progenitor KLS^−^ (cKit^+^Lineage^−^Sca-1^−^) population and the mature Lin^+^ (Lineage^+^) population. We then applied metabolomics techniques to examine the metabolites of these cells. Although quiescent normal HSCs reportedly produce adenosine 5′-triphosphate through anaerobic glycolysis^19^,^20^, we observed no differences in levels of glucose, fructose 1,6-bisphosphate or pyruvate between normal KLS^+^ cells and CML-KLS^+^ cells (Fig. 1a; Supplementary Data 1). Adenosine 5′-monophosphate levels were slightly higher in CML-KLS^+^ cells than in normal KLS^+^ cells, but adenosine 5′-triphosphate was not measurable by this approach in either population.

When we determined levels of various individual amino acids, we observed no differences between normal KLS^+^ and CML-KLS^+^ cells (Fig. 1b; Supplementary Data 1). However, to our surprise, several dipeptide species were markedly increased in CML-KLS^+^ cells compared with normal KLS^+^ cells, whether the latter cells were isolated from healthy littermate (FVB/N) mice (Fig. 1c; Supplementary Fig. 1) or from healthy C57BL/6 control mice at 8 or 24 weeks of age (Supplementary Data 1). A calculation of the ratio of dipeptide levels in CML cells versus normal cells at each stage indicated that, compared with most Lin^+^-differentiated CML cells, it is the immature CML-KLS^+^ population that tends to have the largest dipeptide content (Fig. 1d). While dipeptides were also elevated in the CML-KLS^−^ progenitor population, for reasons elaborated below, we believe that this increase was likely due to the increased protein turnover/degradation that is required to support the vigorous proliferation of CML progenitors. Although we cannot exclude the possibility that there is a systemic increase in dipeptide species in the blood of tet-inducible CML-affected mice, we saw no obvious correlation between dipeptide levels in the rare population of CML-KLS^+^ cells and the vast majority of differentiated CML-Lin^+^ cells (Supplementary Fig. 2). Thus, a mechanism(s) intrinsic to CML stem cells may contribute to their accumulation of dipeptide species *in vivo*. We then set out to determine why, unlike normal HSCs, quiescent CML stem cells store amino acids in dipeptide pools.

### CML stem cells take up dipeptides via the Slc15A2 transporter

**Figure 2:**
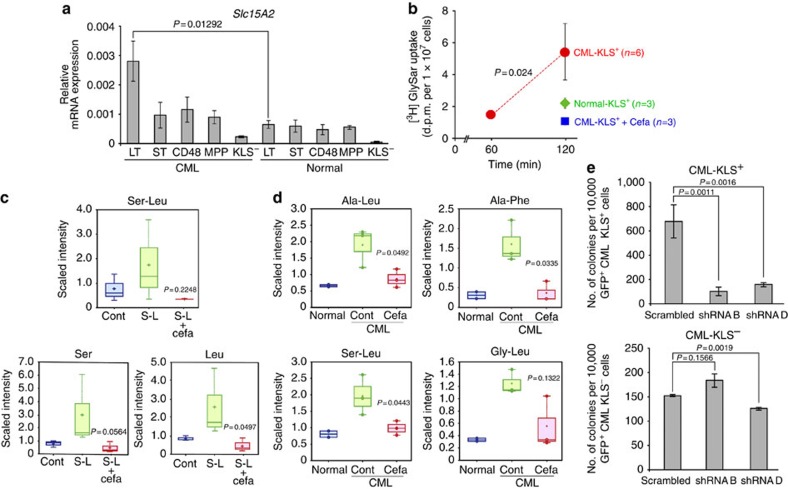
CML stem cells internalize dipeptides via the Slc15A2 dipeptide transporter. (**a**) qRT–PCR determination of relative *Slc15A2* mRNA levels in LT stem, ST stem, CD48^+^, MPP and KLS^−^ cells from CML-affected (*Tal1-tTA*^*+*^*TRE-BCR-ABL1*^*+*^) mice (male, *n*=1; female, *n*=5) and normal littermate (*Tal1-tTA*^*+*^) mice (male, *n*=4; female, *n*=4) at 5 weeks post DOX withdrawal. Data are the mean ratio±s.d. of transcript levels normalized to *Actb* (*n*=3) (*P* value, LT-CML stem cells versus normal LT-HSCs; Student's *t*-test). (**b**) Uptake of [^3^H]GlySar by normal-KLS^+^cells, and CML-KLS^+^cells. Cefadroxil (Cefa), Slc15A2 chemical competitor. Data are the mean d.p.m.±s.d. (*n*=3) (*P* value, Student's *t*-test). (**c**) Uptake of the dipeptide Ser–Leu (S-L) by CML-KLS^+^ cells incubated for 2 h. Internalized S–L and hydrolysed Ser and Leu were determined by metabolomics. Amounts of dipeptides or amino acids were plotted in whisker boxes. Cross, mean value; horizontal line across box, median value; error bars, maximum and minimum of distribution (*P* value, Cont versus Cefa; Welch's *t*-test). (**d**) Metabolomic analyses of dipeptide species in normal-KLS^+^ and CML-KLS^+^ cells isolated from normal littermates (male, *n*=6; female, *n*=6; two experiments), and CML-affected mice that received either vehicle (Cont; male, *n*=2; female, *n*=4; three experiments) or Cefa (male, *n*=0; female, *n*=4; three experiments) for 30 days (*P* value, Cont versus Cefa; Welch's *t*-test). (**e**) Quantification of colony-forming capacity of CML-KLS^+^ and CML-KLS^−^ cells lentivirally transduced to express scrambled shRNA, or shRNA targetting *Slc15A2* (shRNA B and D). GFP^+^ cells were co-cultured on OP-9 stromal cells (3% O_2_) for 5 days. Data are the mean colony number±s.d. (*n*=3) and are representative of three experiments (*P* value, Student's *t*-test).

To investigate why dipeptides accumulate in immature CML cells, we examined upstream gene expression patterns. We isolated the most primitive LT stem cells (CD150^+^CD48^−^CD135^−^KLS^+^ cells)^8^, short-term (ST) stem cells (CD150^−^CD48^−^CD135^−^ KLS^+^ cells) and KLS^−^ progenitor cells (Supplementary Fig. 3) from healthy littermate control and CML-affected mice and performed gene expression profiling using next-generation RNA sequencing. We screened for genes that were upregulated in LT-CML stem cells but not in CML-KLS^−^ cells or normal LT-HSCs, and identified 107 such genes (Supplementary Data 2). Most notable among these was the *Slc15A2* gene encoding an oligo-/dipeptide transporter, which quantitative real-time RT–PCR analyses confirmed was highly expressed in LT-CML stem cells compared with not only CML-KLS^−^ progenitors but also normal LT-HSCs (Fig. 2a; Supplementary Data 2).

To perform a functional analysis of whether Slc15A2 activity was in fact implicated in the observed dipeptide accumulation, we first incubated CML-KLS^+^ cells *in vitro* with [^3^H]-labelled glycylsarcosine (GlySar)^21^,^22^, which is a dipeptide analogue that cannot be metabolized and acts as a substrate of Slc15A family transporters. Interestingly, CML-KLS^+^ cells internalized much more [^3^H]GlySar than did normal KLS^+^ cells, and this uptake was markedly decreased in the presence of the Slc15A2-specific chemical competitor cefadroxil^23^ (Fig. 2b). We next incubated CML-KLS^+^ cells with exogenous dipeptide (Ser–Leu) *in vitro*, followed by metabolomics analysis. Compared with control CML-KLS^+^ cells, CML-KLS^+^ cells incubated with Ser–Leu tended to show increased levels of both the Ser–Leu dipeptide and its hydrolysed component amino acids (Ser and Leu) (Fig. 2c). Importantly, treatment with cefadroxil markedly suppressed these accumulations of Ser–Leu, Ser and Leu, indicating that, even though the internalized dipeptide was rapidly hydrolysed, CML stem cells cultured *in vitro* still possess intrinsic dipeptide transporter activity. We also evaluated the possibility that defective protein degradation might contribute to the dipeptide accumulation in CML stem cells. Treatment of these cells *in vitro* with Bortezomib (a 26S proteasome inhibitor) or Bafilomycin A1 (an autophagy inhibitor) tended to decrease individual amino-acid levels (Supplementary Fig. 4). However, in these same cells, treatment with the inhibitors induced only one instance of statistically significant dipeptide accumulation (Supplementary Fig. 5). Thus, a defect in proteasomal degradation or autophagy does not appear to be the major cause of dipeptide accumulation in CML stem cells.

On the basis of these *in vitro* results, we examined whether cefadroxil could attenuate dipeptide internalization by CML stem cells *in vivo.* CML-affected mice received oral administration of cefadroxil for 30 days, followed by metabolomics analysis of CML stem cells to measure intracellular dipeptides. Intriguingly, exposure of mice to cefadroxil decreased levels of several dipeptides in immature CML-KLS^+^ cells, implying impaired uptake of these dipeptide species (Fig. 2d). Combined with our *in vitro* data, these *in vivo* results implicate Slc15A2 transporter activity as a major driver of dipeptide accumulation in CML stem cells.

To understand the biological role of dipeptide uptake in CML stem cells, we evaluated how inhibition of dipeptide transporter function affected CML stem cell activity *in vitro*. Lentiviral transduction of short hairpin RNAs (shRNAs) targetting *Slc15A2* messenger RNA (mRNA) also decreased the colony-forming capacity of CML-KLS^+^ cells but not that of CML-KLS^−^ cells (Fig. 2e). These data suggest that dipeptide uptake through the Slc15A2 transporter maintains CML stem cell activity *in vitro*.

To identify the pathway mediating intracellular nutrient signalling associated with dipeptide uptake, we first investigated whether treatment *in vitro* of LT-CML stem cells with GlySar or cefadroxil affected signalling via the mTORC1 pathway^24^,^25^. We exposed LT-CML stem cells to 5 μM GlySar or 5 μM cefadroxil for 30 min and used the highly sensitive Duolink *in situ* proximity ligation assay (D-PLA) to evaluate both phosphorylated Raptor-Ser863 and phosphorylated S6 ribosomal protein, which indicate mTORC1 activation^26^. As expected, we found that untreated control LT-CML stem cells exhibited both phospho-Raptor-Ser863 and phospho-S6 (Supplementary Fig. 6). However, after treatment with GlySar or cefadroxil, LT-CML stem cells displayed decreased phosphorylation of Raptor-Ser863 and S6, a result mimicked by treatment with the mTORC1 inhibitor rapamycin (Supplementary Fig. 6). These data indicate that interference with Slc15A2-mediated dipeptide uptake, either by non-metabolizable analogue or chemical competitor, attenuates mTORC1-mediated nutrient signalling in LT-CML stem cells.

AMPK becomes phosphorylated in cells experiencing low energy or nutrient starvation conditions, leading to suppression of the downstream mTORC1 pathway^27^. Treatment of LT-CML stem cells with Metformin^28^, a known activator of AMPK, increases the phosphorylation of both AMPK and Raptor-Ser792, and phospho-Raptor-Ser792 suppresses mTORC1 activity^27^. However, although our treatment of LT-CML stem cells with GlySar or cefadroxil increased phospho-AMPK, these agents did not promote Raptor-Ser792 phosphorylation (Supplementary Fig. 7). Thus, the AMPK is dispensable for the suppression of the mTORC1 pathway seen in LT-CML stem cells experiencing inhibition of Slc15A2-mediated dipeptide uptake.

### Smad3–Ser208 phosphorylation supports LT-CML stem cells

Although we found that dipeptides were able to influence nutrient signalling via the mTORC1 pathway (Supplementary Fig. 6), it has been reported that rapamycin treatment does not prolong the survival of CML-affected mice^29^, suggesting that mTORC1 signalling is not crucial for the maintenance of CML stem cells *in vivo.* Because the TGF-β–FOXO–BCL6 signalling pathway is essential for CML stem cell maintenance *in vivo*^15^,^30^,^31^,^32^, we speculated that there might be a connection between this axis and dipeptide-mediated nutrient signalling that could promote CML stem cell activity *in vivo*.

**Figure 3:**
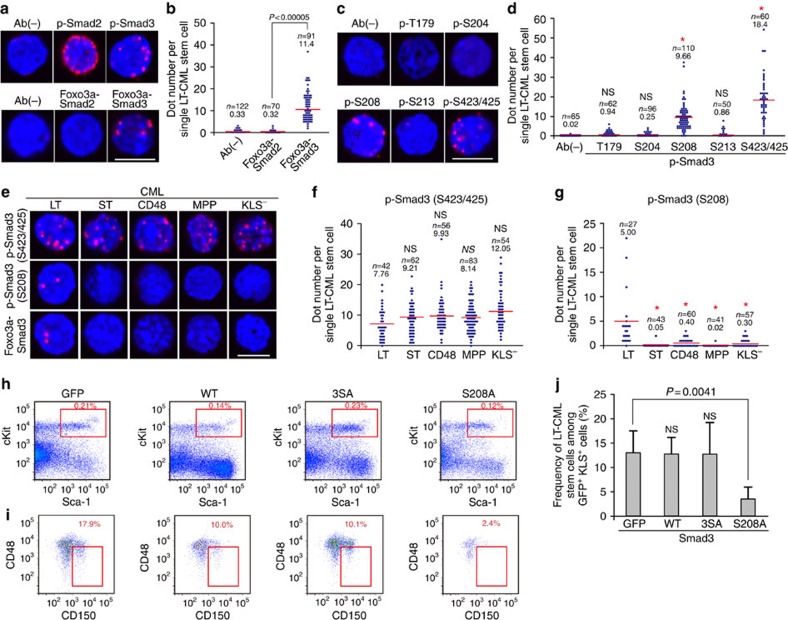
Non-canonical Smad3–Ser208 phosphorylation supports LT-CML stem cells *in vivo.* (**a**) Duolink *in situ* PLA (D-PLA) imaging of (top) Smad2/3 C-terminal phosphorylation and (bottom) interaction between Foxo3a and Smad2 or Smad3 in LT-CML stem cells. The combinations of primary antibodies used are listed in Supplementary Table 1. Ab(−), technical negative control using a single mouse anti-Smad3 antibody. Nuclei were visualized using DAPI. Results are representative of three trials. Scale bar, 10 μm. (**b**) Quantification of dot number per single LT-CML stem cell in the bottom panel of **a** from the three experiments. The mean dot number (red line) appears under total cells number (*n*). *P* value was measured by the Student's *t*-test. (**c**,**d**) D-PLA imaging and quantification of Smad3 phosphorylation at the indicated sites in freshly isolated LT-CML stem cells from the three experiments. Scale bar, 10μm. (*P<0.0005 compared with Ab(−); Student's *t*-test; NS, not significant). (**e**–**g**) D-PLA imaging and quantification of Smad3–Ser423/425 and Smad3–Ser208 phosphorylation, and Foxo3a–Smad3 interaction, in the indicated CML cell subsets, from the three experiments. Scale bar, 10μm. (*P<0.0005 compared with LT-CML stem cell; Student's *t*-test; NS). (**h**,**i**) Flow cytometric quantification of (**h**) the indicated GFP^+^CML-KLS^+^ cell subpopulations (red rectangles) among total GFP^+^CML cells, and (**i**) the most primitive GFP^+^LT-CML stem cells (red rectangles) among GFP^+^CML-KLS^+^ cells, isolated from mice transplanted with CML-KLS^+^ cells bearing retroviral vectors expressing GFP, Smad3-WT, Smad3–3SA or Smad3–S208A. Results are representative of three trials. (**j**) Quantification of the frequency of GFP^+^LT-CML stem cells among the GFP^+^CML-KLS^+^ cells analysed in **i**. Data are the mean percentage of GFP^+^ LT-CML stem cells±s.d. (*n=3*) (*P* value, Student's *t*-test; NS).

To identify the key molecule responsible for the potential cross-talk between nutrient signalling and the TGF-β–FOXO axis, and thereby for CML stemness, we investigated whether Smad2/3, the downstream effectors of TGF-β signalling, were implicated in CML stem cell activity. We found that both Smad2 and Smad3 were phosphorylated at the relevant C-terminal sites in freshly purified LT-CML stem cells (Fig. 3a, top). However, D-PLA analysis revealed that only Smad3 interacted with Foxo3a in these cells, consistent with a previous report^33^ (Fig. 3a, bottom and Fig. 3b). These results suggested that Smad3 might be involved in the TGF-β–FOXO signalling cascade responsible for CML stem cell maintenance. Because Smad3 is a known stemness transcription factor^16^,^34^, it has been of great interest to determine whether Smad3 promotes the maintenance of CML cell stemness.

We next examined Smad3 phosphorylation sites in freshly purified LT-CML stem cells. Whereas D-PLA detected total phosphorylation of Smad3 at Thr179, Ser204, Ser208, Ser213 and Ser423/425 in TGF-β-treated LT-CML stem cells, as expected (Supplementary Fig. 8), freshly purified LT-CML stem cells showed canonical Smad3 phosphorylation at Ser423/425 and non-canonical phosphorylation only at Ser208 (Fig. 3c,d). Interestingly, although Smad3–Ser423/425 was also phosphorylated in ST-CML stem cells and in CD48^+^, multipotent progenitor (MPP; CD135^+^KLS^+^) and KLS^−^ CML cells, Smad3–Ser208 phosphorylation was unique to the most primitive LT-CML stem cells, as was Smad3–Foxo3a interaction (Fig. 3e–g). These data suggest that phosphorylation of Smad3 at Ser208 may allow LT-CML stem cells to activate Foxo3a, whose transcriptional activity supports CML stem cell maintenance *in vivo*^15^.

To determine the biological relevance of Smad3 phosphorylation at Ser423/425 and Ser208, we took advantage of two mutant forms of human Smad3 that cannot be phosphorylated: Smad3–3SA, in which Ser422/423/425 are all converted to Ala, and Smad3–S208A, in which Ser208 is converted to Ala^35^. We infected CML-KLS^+^ cells with retroviral vectors expressing either control green fluorescent protein (GFP), Smad3-wild type (WT), Smad3–3SA or Smad3–S208A, and transplanted these cells into recipient mice (Supplementary Fig. 9). CML stem cell maintenance *in vivo* was then evaluated by flow cytometry. By 30 days post transplantation, the Smad3 mutations had not affected the size of the GFP^+^(Smad3^+^) CML-KLS^+^ cell population (Fig. 3h). However, we found a striking decrease in the frequency of the most primitive LT-CML stem cells in recipients transplanted with CML-KLS^+^ cells expressing Smad3–S208A (Fig. 3i,j). Thus, inhibition of non-canonical Smad3–Ser208 phosphorylation in LT-CML stem cells impairs their maintenance *in vivo*.

### p38MAPK/Smad3/Foxo3a axis maintains CML stem cells

**Figure 4:**
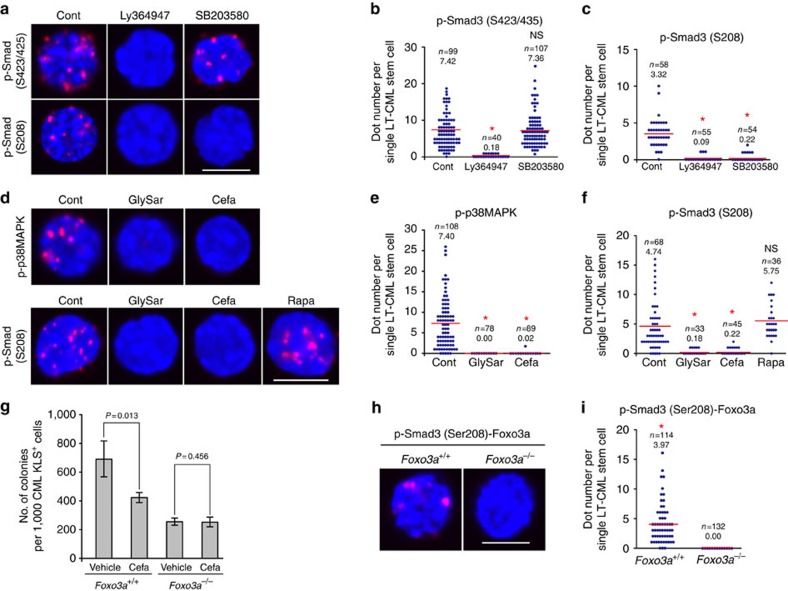
Dipeptide-induced nutrient signalling regulates CML stem cell activity through a p38MAPK/Smad3–Ser208/Foxo3a axis. (**a**–**c**) D-PLA imaging and quantification of (**a**,**b**) Smad3–Ser423/425 and (**a**,**c**) Smad3–Ser208 phosphorylation in LT-CML stem cells treated for 30 min with dimethyl sulfoxide (Cont), Ly364947 (TGF-β type I receptor kinase inhibitor; 5 μM) or SB203580 (p38MAPK inhibitor; 5 μM). Nuclei were visualized using DAPI. Results are representative of three trials. The combinations of primary antibodies used are listed in Supplementary Table 1. Scale bar, 10 μm. (*P* value, Student's *t*-test; NS, not significant.) (**d**–**f**) D-PLA imaging and quantification of (**d**,**e**) phospho-p38MAPK and (**d**,**f**) phospho-Smad3–Ser208 in LT-CML stem cells treated for 30 min with vehicle (Cont), GlySar (5 μM) or cefadroxil (Cefa; 5 μM). Scale bar, 10 μm. (*P<0.00005 compared with cont; Student's *t*-test; NS). (**g**) Quantification of *in vitro* colony-forming capacity of *Foxo3a*^*+/+*^LT-CML stem cells and *Foxo3a*^*−/−*^LT-CML stem cells. Vehicle or Cefa (5 μM). Data are the mean colony number±s.d. (*n*=3) and are representative of three experiments (*P* value, Student's *t*-test). (**h**,**i**) D-PLA imaging and quantification of the interaction between phospho-Smad3–Ser208 and Foxo3a in *Foxo3a*^*+/+*^LT-CML stem cells. *Foxo3a*^*−/−*^LT-CML stem cells were analysed as a negative control. Scale bar, 10μm. (*P<0.00005; Student's *t*-test).

We sought to clarify which upstream signalling pathway mediated the Smad3–Ser208 phosphorylation critical for LT-CML stem cell maintenance. On the basis of the amino-acid sequence surrounding Ser208 in human and mouse Smad3 [PNL**S**(208)PNPM], we used the NetPhosK1.0 server of the Technical University of Denmark ( http://www.cbs.dtu.dk/services/NetPhos/)^36^ to predict the identity of the kinase phosphorylating Smad3–Ser208. Several kinases involved in cell proliferation phosphorylate Smad3 at the linker domain, but p38MAPK is the only kinase validated thus far to phosphorylate Smad3–Ser208, as reported previously^37^. To ascertain whether p38MAPK phosphorylates Smad3–Ser208 in LT-CML stem cells, we employed SB203580, a chemical inhibitor of p38MAPK. Although treatment of LT-CML stem cells with Ly364947, a TGF-β type I receptor kinase inhibitor, blocked phosphorylation of Smad3 at both Ser423/425 and Ser208 (Fig. 4a–c), inhibition of p38MAPK with SB203580 decreased only Smad3–Ser208 phosphorylation (Fig. 4a,c). Cyclin-dependent kinases 8/9 (CDK8/9) also reportedly promote agonist-induced Smad3–Ser208 phosphorylation^38^, but we found that treatment of LT-CML stem cells *in vitro* with the CDK9 inhibitor Flavopiridol did not attenuate Smad3–Ser208 phosphorylation (Supplementary Fig. 10). These data indicate that p38MAPK is a bona fide kinase for Smad3, and link p38MAPK activity to non-canonical Smad3–Ser208 phosphorylation and LT-CML stem cell maintenance.

Given that p38MAPK is regulated by amino-acid signalling^39^, we hypothesized that the increased dipeptide uptake in LT-CML stem cells might trigger activation of a nutrient-associated p38MAPK pathway. We therefore treated LT-CML stem cells with either GlySar or cefadroxil and used D-PLA to assess p38MAPK phosphorylation. Both inhibitors suppressed the phosphorylation of p38MAPK–Thr180/Tyr182 and blocked p38MAPK-mediated phosphorylation of Smad3–Ser208 (Fig. 4d–f). In contrast, treatment with rapamycin did not suppress non-canonical Smad3–Ser208 phosphorylation (Fig. 4d,f). These results indicate that, at least in LT-CML stem cells, dipeptide species can induce activation of nutrient signalling through p38MAPK and drive its downstream phosphorylation of Smad3–Ser208. They further suggest that internalized dipeptides stimulate mTORC1- and p38MAPK-mediated nutrient signalling pathways in parallel.

Because both Smad3–Ser208 phosphorylation and Smad3–Foxo3a interaction were detectable only in LT-CML stem cells (Fig. 3e), we wondered whether the phosphorylation of Smad3–Ser208 might be involved in regulating Foxo3a's recently reported function in CML stem cell maintenance^15^. To investigate this hypothesis, we examined whether the suppression of LT-CML stem cell colony-forming capacity induced by cefadroxil *in vitro* was reduced in *Foxo3a*-disrupted LT-CML stem cells. To establish a *Foxo3a*-deficient CML mouse model, we generated *Foxo3a*^*−/−*^ tet-inducible CML mice and *Foxo3a*^*+/+*^ littermate controls (Methods). We then isolated LT-CML stem cells from *Foxo3a*^*−/−*^ and *Foxo3a*^*+/+*^ CML-affected littermates at 5 weeks post DOX withdrawal. Consistent with our previous report^15^, *Foxo3a*^*−/−*^ LT-CML stem cells exhibited a decrease in colony-forming capacity *in vitro* compared with *Foxo3a*^*+/+*^ LT-CML stem cells (Fig. 4g). However, the number of colonies formed by *Foxo3a*^*−/−*^ LT-CML stem cells was not altered by cefadroxil treatment. Furthermore, D-PLA revealed an interaction between phospho-Smad3–Ser208 and Foxo3a in *Foxo3a*^*+/+*^ LT-CML stem cells that did not occur in *Foxo3a*^*−/−*^ LT-CML stem cells (Fig. 4h,i). Taken together, these results suggest that the p38MAPK/Smad3–Ser208 axis activated by internalized dipeptides maintains CML stem cells in a Foxo3a-dependent manner.

**Figure 5:**
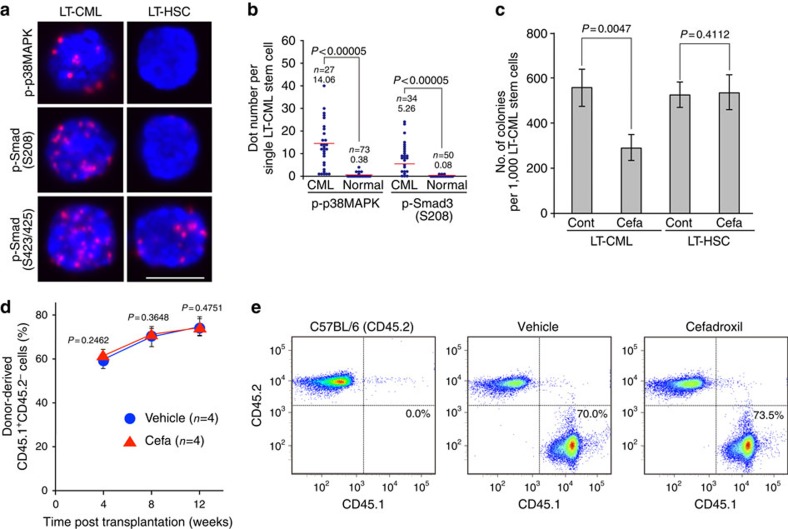
Dipeptide transporter chemical competitor does not inhibit normal HSCs *in vivo*. (**a**,**b**) D-PLA imaging and quantification of p38MAPK–Thr180/Tyr182, Smad3–Ser208 and Smad3–Ser423/425 phosphorylation in LT-CML stem cells and normal LT-HSCs. Nuclei were visualized using DAPI. Results are representative of three trials. The combinations of primary antibodies used are listed in Supplementary Table 1. Scale bar, 10 μm. (*P* value, Student's *t*-test.) (**c**) Quantification of colony-forming capacity of LT-CML stem cells and normal LT-HSCs treated with vehicle (Cont) or Cefa (5 μM) on OP-9 stromal cells (3% O_2_) for 5 days. Data are the mean colony number±s.d. (*n=3*) and are representative of three experiments (*P* value, Student's *t*-test). (**d**,**e**) Competitive reconstitution assay to determine the effects of Cefa on normal HSCs. (**d**) Lethally irradiated (9 Gy) C57BL/6 (CD45.2) recipient mice were reconstituted with 1 × 10^4^ normal KLS^+^ cells (CD45.1) plus 5 × 10^5^ unfractionated BM MNCs (CD45.2). Recipients were treated with vehicle (female, *n*=4) or Cefa (36 mg kg^−1^ per day; female, *n*=4) from day 0 to 12 weeks (wks) post transplantation. Reconstitution by donor-derived cells (CD45.1^+^) was monitored at 4, 8 and 12 wks by flow cytometry. Data are the mean (%) of donor-derived CD45.1^+^CD45.2^−^ cells among PBMCs±s.d. (*n*=4) (*P* value, Student's *t*-test.) (**e**) Representative flow cytometric analyses of donor-derived CD45.1^+^CD45.2^−^cells among the PBMCs at 8 wks in **d** stained with anti-CD45.1 and anti-CD45.2 mAb. Cells from littermate C57BL6 (CD45.2) mice were stained as a negative control.

We next investigated whether dipeptide-stimulated activation of p38MAPK–Smad3 signalling was specific to CML stem cells or also occurred in normal HSCs. Consistent with the minimal dipeptide pools observed in normal HSCs (Fig. 1c,d; Supplementary Figs 1 and 2), phosphorylation of neither p38MAPK–Thr180/Tyr182 nor Smad3–Ser208 was detected in normal LT-HSCs (Fig. 5a,b). In contrast, and consistent with previous reports^14^,^15^,^40^,^41^, Smad3–Ser423/425 phosphorylation occurred in both LT-HSCs and LT-CML stem cells (Fig. 5a). These data confirm the functionality of canonical TGF-β signalling in normal LT-HSCs and LT-CML stem cells, and highlight a dipeptide/p38MAPK-mediated signalling pathway unique to LT-CML stem cells.

We next investigated whether treatment with cefadroxil inhibits the function of CML stem cells and normal HSCs *in vitro*. Treatment of isolated LT-CML stem cells with cefadroxil significantly reduced their colony-forming capacity *in vitro*, whereas cefadroxil-treated LT-HSCs maintained normal levels of colony-forming capacity (Fig. 5c). To confirm that cefadroxil administration *in vivo* does not alter the function of normal HSCs, we employed a well-established competitive reconstitution assay^42^. Irradiated CD45.2 recipient mice were co-transplanted with 1 × 10^4^ purified normal KLS^+^ cells from congenic CD45.1 mice plus 5 × 10^5^ unfractionated BM mononuclear cells (MNCs) from healthy CD45.2 mice. These animals then received daily administration of cefadroxil or vehicle for 12 weeks post transplantation. Importantly, there was no decrease in the frequency of donor-derived CD45.1 MNCs in peripheral blood of recipients after 4, 8 or 12 weeks of cefadroxil administration (Fig. 5d,e). Concomitantly, there was a comparable increase in the degree of chimerism originating from donor-derived normal KLS^+^ cells, whether or not cefadroxil was present (Fig. 5d). Thus, administration of cefadroxil *in vivo* has no detectable effect on the reconstitutive powers of normal HSCs.

### Targetting of dipeptide signalling eradicates CML stem cells

**Figure 6:**
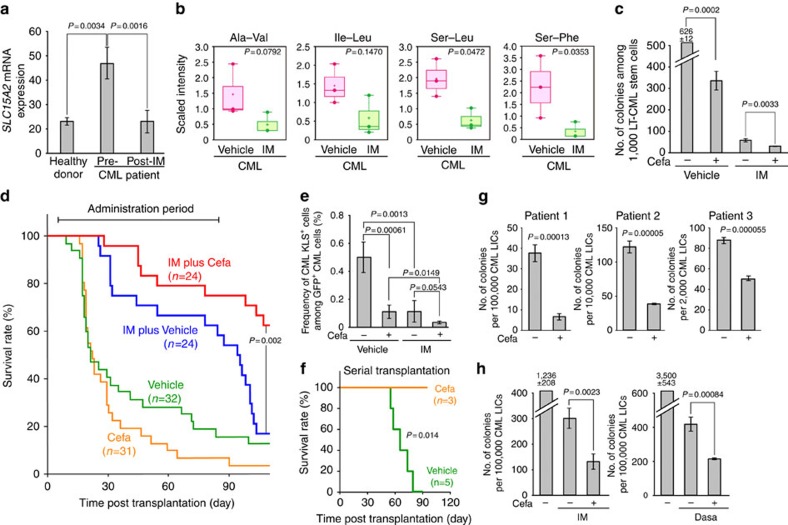
Inhibition of dipeptide uptake in combination with TKI therapy eradicates CML stem cells *in vivo*. (**a**) Relative *SLC15A2* mRNA expression in human CML patients as determined by microarray analysis of nine healthy donors and nine CML patients pre- and post IM therapy. Data are from a public database (GEO, GSE33075) (*P* value, one-sided *t*-test). (**b**) Metabolomic analyses of dipeptides in CML-KLS^+^ cells from CML-affected mice that received either vehicle (male, *n*=2; female, *n*=4; three experiments) or IM (100 mg kg^−1^ per day; male, *n*=2; female, *n*=6; three experiments) for 30 days. Amounts of dipeptides were plotted in whisker boxes. Cross, mean value; horizontal line across box, median value; error bars, maximum and minimum of distribution (*P* value, vehicle versus Cefa; Welch's *t*-test). (**c**) Quantification of colony-forming capacity of LT-CML stem cells (3% O_2_) with either vehicle (−) or 5 μM Cefa (+) for 5 days in the absence or presence of 1 μM IM. Data are the mean colony number±s.d. (*n*=3) and are representative of three experiments (*P* value, Student's *t*-test). (**d**) Survival rates of CML-affected mice (female) that received vehicle, IM (100 mg kg^−1^ per day) and/or Cefa (36 mg kg^−1^ per day) for days 8–90 post transplantation. Results shown are cumulative data obtained from three independent experiments. Statistical differences were determined using the log-rank non-parametric test. (**e**,**f**) Eradication of CML stem cells *in vivo* by cefadroxil. CML-affected mice received vehicle (−), IM and/or Cefa (+) daily for 30 days post transplantation as in **d**. (**e**) Mean frequency±s.d. of GFP/BCR-ABL1^+^CML-KLS^+^cells among total GFP/BCR-ABL1^+^CML cells (*n*=3) (*P* value, Student's *t*-test). (**f**) Survival rate of new recipient mice (female) that received serial transplantation of GFP/BCR-ABL1^+^CML-KLS^+^cells (3 × 10^4^ cells) from the CML-affected mice in **e** that had been treated with vehicle (female, *n*=4) or Cefa (female, *n*=5). Mouse survival was monitored for 90 days (*P* value, log-rank non-parametric test). (**g**,**h**) Quantification of colony-forming capacity of human CD34^+^CD38^−^Lin^−^CML-LICs that were treated *in vitro*: (**g**) with either vehicle (−) or 5 μM Cefa (+) for 5 days, or (**h**) with vehicle (−) or 5 μM Cefa (+) in the absence or presence of 1 μM IM or 500 nM dasatinib (Dasa) for 3 days. Data shown are the mean colony number±s.d. (*n*=3) (*P* value, Student's *t*-test).

The specificity of dipeptide-induced nutrient signalling to LT-CML stem cells prompted us to investigate whether this pathway might be a possible therapeutic target; that is, whether disruption of dipeptide internalization might lead to the eradication of CML stem cells and a reduction in disease relapse. First, to determine whether the *SLC15A2* gene is upregulated in human CML patients as it is in CML-affected mice, we retrieved data on levels of *SLC15A2* mRNA in cells of CML patients listed in a public database gene expression omnibus (GEO: GSE33075). Intriguingly, prior to IM therapy, *SLC15A2* mRNA levels were indeed higher in BM leukaemia cells of nine CML patients than in the BM haematopoietic cells of nine healthy individuals (Fig. 6a). However, after IM therapy, *SLC15A2* mRNA levels in the same nine CML patients had decreased to a level comparable to that in healthy individuals. To further explore this finding, we returned to our mouse model and compared dipeptide levels in CML-KLS^+^ cells isolated from CML-affected mice that had received vehicle or IM therapy for 1 month. In line with our observations in human CML patients, metabolomic analysis of these mice indicated that IM treatment tended to decrease dipeptide levels in CML-KLS^+^ cells (Fig. 6b). In addition, IM treatment of murine LT-CML stem cells *in vitro* reduced levels of phospho-p38MAPK and phospho-Smad3–Ser208 (Supplementary Fig. 11). These results suggest that the accumulation of dipeptide species may not be the direct cause of TKI-resistance in the CML stem cell population responsible for disease recurrence. However, our findings also suggest that the SLC15A2-mediated nutrient supply we have identified plays a critical role in human CML leukaemogenesis.

However, we cannot exclude the possibility that an unidentified minor population of CML stem cells that is responsible for IM resistance accumulates high levels of dipeptides, and that it is this subset that drives the subsequent clonal evolution of CML stem cells post IM therapy *in vivo*. Thus, we next evaluated the potential therapeutic benefit of combined administration of a TKI, which blocks the activity of BCR-ABL1 kinase, with cefadroxil, which inhibits Slc15A2-mediated nutrient signalling. When we cultured murine LT-CML stem cells *in vitro* with cefadroxil plus IM, colony formation was reduced compared with treatment with IM alone (Fig. 6c). When we then examined this approach *in vivo*, we found that treatment of CML-affected mice with IM alone delayed disease onset compared with the vehicle-treated group, but, as expected, these animals eventually experienced recurrence of *BCR-ABL1*^*+*^ disease after discontinuation of the therapy (Fig. 6d, blue line). Curiously, the administration of cefadroxil alone appeared to promote disease development (Fig. 6d, orange line). However, the combined administration of IM plus cefadroxil significantly reduced the recurrence rate of *BCR-ABL1*^*+*^ disease compared with the group treated with IM alone (Fig. 6d, red line).

We then determined whether cefadroxil administration could in fact eradicate primitive CML stem cells in CML-affected mice *in vivo*. Indeed, the frequency of CML-KLS^+^ cells among GFP/BCR-ABL1^+^CML cells isolated from BM of CML-affected mice was significantly decreased by cefadroxil exposure *in vivo* (Fig. 6e; Supplementary Fig. 12). Although IM alone also reduced the frequency of CML-KLS^+^ cells, the combined administration of IM plus cefadroxil had a much greater repressive effect on this population (Fig. 6e; Supplementary Fig. 12). Notably, in serial transplantation experiments, CML-KLS^+^ cells isolated from cefadroxil-treated CML-affected mice completely lost their ability to drive *BCR-ABL1*^*+*^ disease in new recipients, allowing the animals to survive for over 90 days (Fig. 6f). In contrast, all mice that received CML-KLS^+^ cells from vehicle-treated CML-affected animals developed *BCR-ABL1*^*+*^ disease and were dead before 80 days, demonstrating that the untreated CML-KLS^+^ cells had retained their CML-initiating ability. These results indicate that oral administration of cefadroxil to inhibit dipeptide uptake may block nutrient signalling important for the maintenance of CML stem cells *in vivo*, and further suggest that cefadroxil used in combination with a TKI can improve the survival of CML-affected mice by eradicating CML stem cells.

To investigate the relevance of our findings to human CML therapy, we evaluated the effects of cefadroxil treatment *in vitro* on CML-LICs obtained from human chronic phase CML patients. We isolated CD34^+^CD38^−^Lin^−^ CML-LICs from BM MNCs of three CML patients and treated these cells *in vitro* with cefadroxil. As expected, cefadroxil suppressed the colony-forming capacity of all three human CML-LIC samples *in vitro* (Fig. 6g). The co-treatment of human CML-LICs with a combination of a TKI (IM or dasatinib) plus cefadroxil reduced colony formation over the suppressive effect of the TKI alone (Fig. 6h).

**Figure 7:**
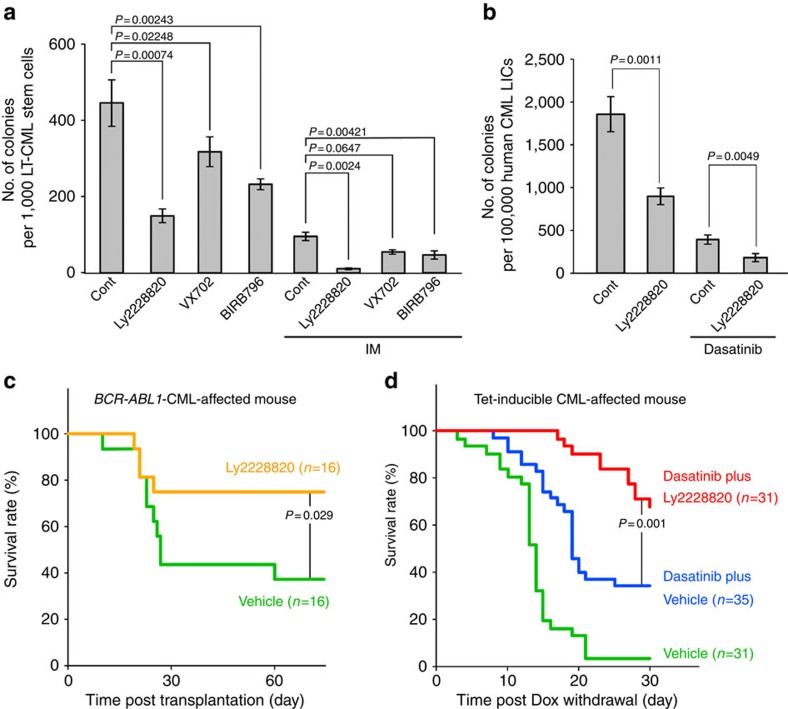
p38MAPK inhibitor depletes CML *in vivo*. (**a**) Quantification of colony-forming capacity of murine LT-CML stem cells treated with DMSO (Cont), the p38MAPK inhibitors Ly2228820 (5 μM), VX-702 (5 μM) or BIRB796 (5 μM) in the absence or presence of IM (1 μM) on OP-9 stromal cells (3% O_2_) for 5 days. Data are the mean colony number±s.d. (*n*=3) (*P* value, Student's *t*-test). (**b**) Quantification of colony-forming capacity of human CML-LICs treated with DMSO (Cont) or Ly2228820 (5 μM) in the absence or presence of dasatinib (500 nM) on OP-9 stromal cells (3% O_2_) for 3 days. Data are the mean colony number±s.d. (*n*=3) (*P* value, Student's *t*-test). (**c**) Survival curve of CML-affected mice (*BCR-ABL1*-CML-affected mice) receiving Ly2228820 alone. CML-affected mice received vehicle (female, *n*=16) or Ly2228820 (2.5 mg kg^−1^ every 3rd day; female, *n*=16). Mouse survival was monitored for up to 60 days. Results shown are cumulative data obtained from two independent experiments (*P* value, log-rank non-parametric test). (**d**) Survival curve of tet-inducible CML-affected mice receiving Ly2228820 plus dasatinib. At 1 day post DOX withdrawal, tet-inducible CML-affected mice received either vehicle or dasatinib (5 mg kg^−1^ per day). At 8 days post DOX withdrawal, these animals received additional vehicle alone (male, *n*=15; female, *n*=16), dasatinib plus vehicle (male, *n*=19; female, *n*=16) or dasatinib plus Ly2228820 (2.5 mg kg^−1^ every 3rd day; male, *n*=12; female, *n*=19). Survival was monitored for up to 30 days. Results shown are cumulative data obtained from five independent experiments (*P* value, log-rank non-parametric test).

Finally, in the same vein, we evaluated whether combined treatment of IM plus one of three clinical grade p38MAPK inhibitors could suppress LT-CML stem cell colony formation *in vitro*. The p38MAPK inhibitors Ly2228820 (ralimetinib)^43^, VX-702 (ref. 44) and BIRB796 (doramapimod)^45^ have already been approved by the FDA (USA) as candidate anti-inflammatory or anti-cancer drugs for patients with rheumatoid arthritis, peripheral artery disease or ovarian cancer. When we treated LT-CML stem cells *in vitro* with any of these drugs, their colony-forming capacity was significantly decreased (Fig. 7a). The colony-forming capacity of human CML-LICs was also suppressed by treatment *in vitro* with the p38MAPK inhibitor Ly2228820 (Fig. 7b). Consistent with this finding, *in vivo* treatment of CML-affected mice with Ly2228820 alone improved their survival rate (Fig. 7c). Furthermore, combined administration of Ly2228820 plus dasatinib delayed disease onset in CML-affected mice compared with administration of dasatinib alone (Fig. 7d). Thus, p38MAPK inhibitors may also be candidate agents capable of targetting CML stem cells.

Collectively, our results indicate that nutrient signalling through a p38MAPK–Smad3 axis that is activated by internalized dipeptide species is essential for CML stem cell activity *in vitro* and *in vivo.* Thus, this nutrient supply and its downstream signalling pathway may offer novel candidate therapeutic targets for eradicating CML stem cells. Our data suggest that inhibitors of this pathway used in combination with TKI therapy may provide concrete clinical benefits for CML patients.

## Discussion

Although recent technical advances have opened up new ways of investigating metabolites in proliferating mature cancer cells, the metabolic pathways allowing rare cancer stem cells to survive when mature cancer cells cannot are still unknown. In this study, we found that CML stem cells effectively employ dipeptide uptake mediated by Slc15A2 activity to guarantee an alternative nutrient supply and maintain LT survival *in vivo*.

**Figure 8:**
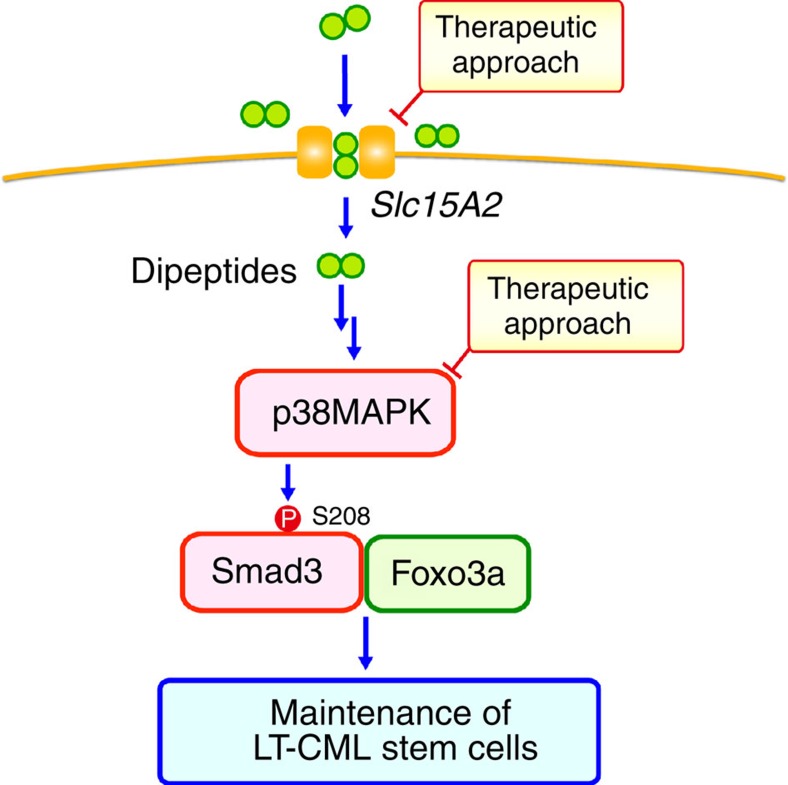
Therapeutic approach based on disruption of a nutrient supply essential for CML stem cells. Diagram outlining the proposed role of dipeptide uptake in LT-CML stem cell maintenance. Dipeptide species internalized by the Slc15a2 dipeptide transporter may initiate nutrient signalling activating p38MAPK. This p38MAPK activation mediates non-canonical Smad3–Ser208 phosphorylation that allows the LT-CML stem cells to activate Foxo3a and is critical for LT-CML stem cell maintenance. Because this mechanism is not important for normal HSCs, interference with dipeptide uptake nutrient signalling may represent a novel therapeutic approach for CML patients.

The results of our study suggest the following model (Fig. 8): (1) CML stem cells accumulate dipeptide species through the Slc15A2 dipeptide transporter; (2) these internalized dipeptides furnish a nutrient source that leads to the activation of nutrient signalling, including signalling via p38MAPK; (3) p38MAPK-mediated non-canonical phosphorylation of Smad3–Ser208 supports Foxo3a's known function in supporting CML stem cell activity *in vivo*. Because normal HSCs do not use this dipeptide-induced nutrient signalling pathway, therapeutic suppression of this novel mechanism should not affect normal HSCs. However, it remains unclear how CML stem cells sense the internalized dipeptides, store them intracellularly and use them as an amino-acid source. For example, protein degradation and/or autophagy pathways may generate dipeptides *en route* to building up supplies of recycled amino acids. It should also be noted that, although we could not detect an obvious increase in amino acids in CML-KLS^+^ cells, including in branched-chain amino acids such as Leu (Fig. 1b; Supplementary Data 1), we cannot exclude the possibility that the downstream nutrient signalling induced by dipeptide uptake includes regulation of the mTORC1 pathway by the cell's amino-acid-sensing machinery^46^,^47^,^48^,^49^,^50^,^51^. Finally, we should clarify how systemic dipeptide levels in the blood affect CML stem cell activity *in vivo*. Further investigation should reveal precisely how CML stem cells use dipeptides to ensure LT-CML stem cell maintenance *in vivo*.

To our knowledge, our study is the first to demonstrate that CML stem cell activity depends on nutrient signalling that regulates post-translational phosphorylation of Smad3 at Ser208. Furthermore, we have shown that dipeptide-induced p38MAPK activation is responsible for this Smad3–Ser208 phosphorylation, and that Foxo3a binds specifically to Smad3 in LT-CML stem cells. Foxo3a is known to cooperate with Smad3 in regulating transcription^33^ and to be required for CML stem cell maintenance^15^,^32^. It has also been reported that p38MAPK interacts with Foxo3a and regulates its function^52^, suggesting a link between the p38MAPK/Smad3–Ser208 axis activated by nutrient signalling and the Foxo3a-mediated pathway supporting CML stem cell activity. Once again, future work should determine how Smad3 interacts with Foxo3a, how phospho-Smad3–Ser208 regulates Foxo3a's activity and whether this interaction regulates the maintenance of CML stemness *in vivo*.

Our results also reveal that the antibiotic cefadroxil attenuates dipeptide uptake by CML stem cells and so may be a promising partner in combination therapy with TKIs. However, we still do not understand how dipeptides act as a nutrient source for specific cell types, or how systemic dipeptide distribution contributes to normal health. Indeed, administration of cefadroxil in the absence of a TKI appeared to accelerate disease development in CML-affected mice. Thus, any application of transporter inhibitors to CML patients must be carefully considered. Nevertheless, we observed elevated dipeptide uptake only in CML stem cells and not in normal HSCs, and cefadroxil combined with TKI decreased dipeptide uptake and survival of CML stem cells but did not affect normal HSCs (Figs 6e,f and 5d,e). Thus, therapeutic inhibition of dipeptide-induced nutrient signalling may eradicate LT-CML stem cells *in vivo* with minimal side effects on normal haematopoiesis. Cefadroxil (Duricef; approved by FDA, USA) has long been safely used as a traditional first-generation cephalosporin antibiotic in humans. Our proposed approach of using cefadroxil to shut down a key nutrient source specific to CML stem cells, in combination with TKI therapy, may thus bring concrete therapeutic benefits to CML patients.

## Methods

### CML mouse models

Several different mouse models of CML-like disease were employed in this study. First, we used a well-described tetracycline (tet)-inducible CML mouse model^8^,^9^,^10^,^18^,^19^. *Tal1-tTA* mice (JAX, #006209)^18^ and *TRE-BCR-ABL1* transgenic mice (JAX, #006202)^17^, both of the FVB/N genetic background, were purchased from the Jackson Laboratory. *Tal1-tTA* and *TRE-BCR-ABL1* transgenic mice were interbred to generate *Tal1-tTA*x*TRE-BCR-ABL1* double-transgenic mice. These animals were maintained in cages supplied with drinking water containing 20 mg l^−1^ DOX (Sigma-Aldrich). At 5–8 weeks after birth, expression of the *BCR-ABL1* oncogene was induced by replacing the DOX-containing drinking water with normal drinking water. Consistent with a previous report^8^, CML-like disease developed in the double-transgenic mutants about 2–5 weeks after DOX withdrawal as in Fig. 7d. These animals were designated as ‘tet-inducible CML-affected mice'.

To establish our *Foxo3a*-deficient tet-inducible CML mouse model, we crossed *Foxo3a*-deficient mice^42^ (C57BL/6; F5) with *Tal-tTA* and *TRE-BCR-ABL1* transgenic mice that were backcrossed for five generations in the C57BL/6 background, respectively.

*BCR-ABL1* transduction/transplantation-based CML model (*BCR-ABL1*-CML mice) has also been used in this study^15^. Briefly, normal KLS^+^ cells (4–5 × 10^3^ cells per recipient mouse) isolated from BM MNCs of 6–8-week-old female mice were transduced with the human *BCR-ABL1-ires GFP* retrovirus and transplanted into irradiated (9 Gy) recipient C57BL/6 mice (female; Sankyo-Lab Service, Tsukuba, Japan). CML-like disease developed in these recipients by 12–20 days post transplantation.

To examine the *in vivo* effects of the combined administration of IM plus cefadroxil, *BCR-ABL1*-CML-affected mice received vehicle alone (artificial gastric fluid solution (900 ml ddH_2_O containing 2.0 g NaCl, 7 ml conc. HCl and 3.2 g pepsin)), or IM (Gleevec; 100 mg kg^−1^ per day; Novartis) and/or cefadroxil (36 mg kg^−1^ per day; Sigma-Aldrich) in vehicle. Treatment was delivered by oral gavage on days 8–90 post transplantation. To examine the effect of Ly2228820 administration alone, *BCR-ABL1*-CML-affected mice received vehicle, or Ly2228820 (2.5 mg kg^−1^ every 3rd day; Axon Medchem) in vehicle, by oral gavage on days 8–60 days post transplantation. To examine the effect of combined administration of dasatinib plus Ly2228820, tet-inducible CML-affected mice received vehicle alone, or dasatinib (5 mg kg^−1^ per day; Brystol-Myers Squibb) in vehicle on days 0–30 post DOX withdrawal, and/or Ly2228820 (2.5 mg kg^−1^ every 3rd day; Axon Medchem) in vehicle on days 7–28 post DOX withdrawal by oral gavage. All animal care in our laboratory was in accordance with the guidelines for animal and recombinant DNA experiments of Kanazawa University.

### Cell sorting

BM MNCs were isolated from the two hindlimbs of 8–12-week-old (male and female) tet-inducible CML-affected mice (*Tal1-tTA*^*+*^ *TRE-BCR-ABL1*^*+*^) and 8–12-week-old (male and female) normal healthy littermate mice (*Tal1-tTA*^+^) at 5 weeks after DOX withdrawal^8^. BM MNCs were first blocked by incubation with anti-FcγIII/II receptor(2.4G2) antibody (Ab) (BD Biosciences), and then stained with anti-Sca-1(E13–161.7)-PE, anti-CD4(L3T4)-FITC, anti-CD8(53-6.7)-FITC, anti-B220(RA3–6B2)-FITC, anti-TER119(Ly-76)-FITC, anti-Gr-1(RB6–8C5)-FITC, and anti-Mac1(M1/70)-FITC (BD Biosciences), anti-CD48(HM48-1)-APC-Cy7 and anti-CD150/SLAM(TC15-12F12.2)-Pacific blue (BioLegend), and anti-cKit(ACK2)-APC and anti-CD135/Flk2/Flt3(A2F10)-biotin (eBiosciences) Abs. Biotinylated primary Abs were visualized using Streptavidin-PE-Cy7 (BD Biosciences).

For metabolomics analysis, a FACS Aria III cell sorter (BD Biosciences) was used to sort immunostained cells into fractions containing immature KLS^+^ (cKit^+^Lineage^−^Sca-1^+^) cells, progenitor KLS^−^ (cKit^+^Lineage^−^Sca-1^−^) cells and differentiated Lin^+^ (Lineage^+^) cells according to a published classification system^15^. For next-generation RNA sequencing and Duolink *in situ* PLA analysis (see below), we further purified KLS^+^ cells into the most primitive LT stem cells (CD150^+^CD48^−^CD135^−^KLS^+^), ST stem cells (CD150^−^CD48^−^CD135^−^KLS^+^), CD48^+^ cells (CD48^+^CD135^−^KLS^+^) and multipotent progenitor-like (MPP) cells (CD135^+^KLS^+^)^8^, as indicated in Supplementary Fig. 3.

For retroviral and lentiviral transductions (see below), CML-KLS^+^ cells and CML-KLS^−^ were purified from BM MNCs of the 8–12-week-old tet-inducible CML-affected mice (male and female). For HSC-competitive reconstitution assays (see below), normal KLS^+^ cells were purified from BM MNCs of 6-week-old C57BL6 congenic (CD45.1) mice (female).

For serial transplantation of CML stem cells (see below), total BM MNCs isolated from the two hindlimbs of *BCR-ABL1*-CML-affected mice were immunostained with anti-Sca-1(E13–161.7)-PE, anti-CD4(L3T4)-biotin, anti-CD8(53-6.7)-biotin, anti-B220(RA3–6B2)-biotin, anti-TER119(Ly-76)-biotin, anti-Gr-1(RB6–8C5)-biotin, anti-Mac1(M1/70)-biotin Abs (BD Biosciences) and anti-c-Kit(ACK2)-APC Ab (eBiosciences). Biotinylated primary Abs were visualized with Streptavidin-PE-Cy7 (BD Biosciences). GFP/BCR-ABL1^+^CML-KLS^+^ cells were purified using a FACS Aria III cell sorter (BD Biosciences).

### Metabolomics

For metabolomic profiling, we isolated 1.8–2.5 × 10^5^ immature KLS^+^ cells, KLS^−^ progenitor cells and Lin^+^-differentiated cells from 8- to 10-week-old CML-affected *Tal1-tTA*^+^ *TRE-BCR-ABL1*^+^ mice (*n*=4 mice in each of the three independent experiments; male and female) and 8- to 10-week old normal healthy littermate control (*Tal1-tTA*^+^) mice (*n*=6 mice in each of the two independent experiments; male and female) at 5 weeks after DOX withdrawal as described above (Supplementary Data 1, study 1). We also determined metabolites in 1.0–1.8 × 10^5^ immature KLS^+^ cells isolated from C57BL/6 mice at 8 and 24 weeks of age (*n*=6 mice in each of the two independent experiments; male and female) (Supplementary Data 1, study 2, age).

To detect inhibition of dipeptide uptake *in vivo*, we isolated immature KLS^+^ cells from 12- to 14-week-old healthy littermate control (*Tal1-tTA*^+^) mice (*n*=6 mice in each of the two independent experiments; male and female) and from 12- to 14-week-old tet-inducible CML-affected (*Tal1-tTA*^*+*^*TRE-BCR-ABL1*^*+*^) mice that had received vehicle (*n*=2 mice in each of the three independent experiments; male and female) or cefadroxil (36 mg kg^−1^ per day; *n*=1–2 mice in each of the three independent experiments; female) by oral gavage for 30 days. For IM administration *in vivo*, we isolated immature KLS^+^ cells from CML-affected mice that had received vehicle (*n*=2 in each of the three independent experiments; male and female) or IM (100 mg kg^−1^ per day; *n*=2–3 in each of the three independent experiments; male and female) by oral gavage for 30 days. For inhibition of protein degradation/turnover *in vitro*, we plated 1.8–2.0 × 10^5^ CML-KLS^+^ cells from CML-affected mice (*n*=8 mice in each of the three independent experiments; male and female) in serum-free SF-03 stem cell medium (Sanko Junyaku) under hypoxic (3% O_2_) conditions and treated them for 2 h with vehicle, 100 nM Bortezomib (Cell Signaling, #2204) or 100 nM Bafilomycin A1 (Sigma, B1793). In all cases, cell pellets were frozen at −80 °C immediately after centrifugation. Metabolomic profiling was performed by Metabolon Inc. (Durham, NC) using ultrahigh-performance liquid chromatography/mass spectroscopy (UPLC/MS/MS) and gas chromatography/MS (GC/MS). Data were compiled using the Metabolon LIMS (Laboratory Information Management System). The UPLC/MS/MS portion of the platform was based on a Waters Acquity UPLC (Waters) and a Q-Exactive high-resolution/accurate mass spectrometer (Thermo Scientific) interfaced with a heated electrospray ionization (HESI-II) source and Orbitrap mass analyzer. GC/MS was performed by a Trace DSQ fast-scanning single-quadrupole mass spectrometer using electron impact ionization (Thermo-Finnigan).

### Next-generation RNA sequencing

LT stem cells, ST stem cells and KLS^−^ progenitor cells isolated from total normal haematopoietic cells, as well as CML cell subsets defined as in Supplementary Fig. 3, were directly sorted into 200 μl Isogene (Nippon Gene) solution. RNA extraction and sequencing were performed by Hokkaido System Science Co. Ltd (Sapporo, Japan). RNA quality was confirmed using Nanodrop (Thermo Fisher Scientific) and an Agilent 2100 Bioanalyzer (Agilent Technologies). All RNA samples had an RNA integrity number of >8.5 and exceeded the quality threshold for RNA sequencing. Libraries were constructed from total RNA using the SMARTer Ultra Low Input RNA kit for Illumina Sequencing (Takara Clontech). RNA was chemically fragmented and converted into single-strand complementary DNA (cDNA) using oligo-dT priming. Paired-end reads of 100 bases were generated using HiSeq2000 (Illumina). Sequence reads in FastQ format were assessed for quality using FastQC. Sequences were mapped to a mouse genome reference (*Mus musculus*; *mm9*, NCBI Build 37) using SeqNova CS by DNAnexus Inc. (Mountain View, CA) ( https://dnanexus.com/). The next-generation RNA-sequencing data for LT-HSCs and LT-CML stem cells were available from a public database gene expression omnibus (GEO, ID: GSE70031) in NCBI, NIH, USA ( http://www.ncbi.nlm.nih.gov/geo/info/linking.html) (Supplementary Data 2).

### Quantitative real-time RT–PCR analysis

Using the RNeasy kit (Qiagen), RNA samples were purified from 4–5 × 10^4^ LT stem cells, ST stem cells, CD48^+^KLS^+^ cells, MPP and KLS^−^ progenitor cells (as defined in Supplementary Fig. 3) isolated from 8- to 10-week-old tet-inducible CML-affected (*Tal1-tTA*^*+*^*TRE-BCR-ABL1*^*+*^; male and female) mice (*n=*6) and 8- to 10-week-old littermate control (*Tal1-tTA*^*+*^; male and female) mice (*n=8*) at 5 weeks post DOX withdrawal. RNA samples were reverse transcribed using the Advantage RT-for–PCR kit (Takara Clontech). Real-time quantitative PCR was performed using SYBR green Premix EX Taq (Takara) on an Mx3000P Real-time PCR system (Stratagene). The following primers were used: 5′-GCATCGCCTTCATTGTGGTG-3′ and 5′-GAGGCTGCTGAAGGCATGGTA-3′ for *Pept2* (*Slc15A2)*; 5′-AGGTCATCACTATTGGCAACGA-3′ and 5′-CACTTCATGATGGAATTGAATGTAGTT-3′ for *Actb* (β-actin). The following cycle parameters were used: denaturation at 95 °C for 10 s, and annealing and elongation at 60 °C for 30 s for *Pept2/Slc15A2* and 58 °C for 30 s for *Actb.*

### Slc15A2 transporter activity

Slc15A2 transporter activity was determined using a well-established assay measuring [^3^H]GlySar uptake by cells suspended in an acidic transport medium (pH 6.0)^21^,^22^. [^3^H]GlySar (28.3 μCi nmol^−1^; MT-1545) was purchased from Moravek Biochemicals (Brea, CA). Briefly, normal KLS^+^ cells or CML-KLS^+^ cells (1 × 10^5^) in 10 μl SF-03 stem cell medium were suspended in 310 μl [^3^H]GlySar (178.57 nmol l^−1^) transport medium (125 mM NaCl, 4.8 mM KCl, 5.6 mM D-glucose, 1.2 mM CaCl_2_–2H_2_O, 1.2 mM KH_2_PO_4_, 1.2 mM MgSO_4_–7H_2_O and 25 mM MES, pH 6.0) in the absence or presence of 100 μM cefadroxil (Sigma-Aldrich) to initiate the transporter reaction. After 60 or 120 min, the radioactivity of [^3^H]GlySar internalized by cells was measured using a liquid scintillation counter.

To examine the uptake of exogenous dipeptide, CML-KLS^+^ cells (1 × 10^5^) were incubated with 1 mM Ser–Leu (H-Ser–Leu-OH, cat. no. 2554151, Kokusan Chemical, Tokyo, Japan) in an acidic transport medium (pH. 6.0) in the absence or presence of cefadroxil (100 μM, Sigma-Aldrich) for 2 h under hypoxic (3% O_2_) conditions at 37 °C. The treated cells were then washed four times with SF-03 medium, centrifuged and the cell pellets were immediately frozen at −80 °C. The internalized dipeptide (Ser–Leu) and its hydrolysed component amino acids (Ser and Leu) were determined by metabolomic profiling performed by Metabolon Inc., as described above.

### cDNA construction and retrovirus preparation

Retroviral expression vectors encoding human Smad3-WT, Smad3–3SA (Ser422, Ser423 and Ser425 all converted to Ala) and Smad3–S208A (Ser208 converted to Ala) were constructed using human WT Smad3 cDNA (kindly provided by Dr Anita B. Roberts, NCI, NIH, Bethesda, MD) as a template^53^. Briefly, cDNAs encoding the Smad3–3SA and Smad3–S208A mutants were constructed in pCR2-TOPO vector (Invitrogen) using the High-Fidelity DNA polymerase KOD Plus 2 kit (Toyobo) or the QuikChange site-directed mutagenesis kit (Stratagene). DNA sequences were confirmed by Operon Biotechnology (Tokyo, Japan) using an ABI-3730xl instrument (Applied Biosystems). EcoRI/XhoI-digested cDNA fragments were inserted into the retroviral expression vector MSCV-*ires*-GFP^15^. Retroviral packaging cells (Plat-E) were transiently transfected with control GFP vector (MSCV-*ires*-GFP) or with MSCV-*ires*-GFP-Smad3-WT, MSCV-*ires*-GFP-Smad3–3SA or MSCV-*ires*-GFP-Smad3–S208A plasmids using FuGene6 (Roche)^15^. At 2 days post transfection, culture supernatants were passed through a 0.45-μm filter and centrifuged at 6,500*g* for 16 h. The virus-containing pellets were resuspended in serum-free SF-03 stem cell medium (Sanko Junyaku) containing 0.1% BSA (#09300; Stem Cell Technology) and penicillin/streptomycin (Gibco), yielding the retroviral solutions used for KLS^+^ cell infection (see below).

### Retroviral infection of KLS^+^ cells and mouse transplantation

Immature CML-KLS^+^ cells were purified from BM MNCs acquired from the two hindlimbs of tet-inducible CML-affected mice as described above. These cells were cultured overnight in a 3% O_2_ incubator at 37 °C in 96-well plates containing 200 μl SF-03 stem cell medium supplemented with 100 ng ml^−1^ human thrombopoietin (TPO, PeproTech) and 100 ng ml^−1^ mouse stem cell factor (SCF, Wako Pure Chemical). The next day, these cells were transferred to 96-well plates pre-treated with retronectin (Takara Bio) and incubated for 30 min with 150 μl of the retroviral solutions described above using Combimag (OZ Biosciences) on a magnetic plate (OZ Biosciences). The top half of the supernatant was carefully removed and the infected cells received an additional 100 μl fresh SF-03 stem cell medium supplemented with TPO and SCF. Infected cells were cultured overnight in a 3% O_2_ incubator at 37 °C. Retrovirally infected CML-KLS^+^ cells (∼1.0–1.5 × 10^5^ cells per recipient mouse) were injected intravenously into lethally irradiated (9.0 Gy) 6-week-old FVB recipient mice (female).

### LT-CML stem cell maintenance *in vivo*

The *in vivo* maintenance of LT-CML stem cells in recipient mice was evaluated at 30 days post transplantation. Total MNCs isolated from the BM of transplant recipients were immunostained with anti-Sca-1(E13–161.7)-PE, anti-CD4(L3T4)-biotin, anti-CD8(53-6.7)-biotin, anti-B220(RA3–6B2)-biotin, anti-TER119(Ly-76)-biotin, anti-Gr-1(RB6–8C5)-biotin, anti-Mac1(M1/70)-biotin Abs (BD Biosciences), anti-CD135/Flk2/Flt3(A2F10)-biotin and anti-c-Kit(ACK2)-APC Abs (eBiosciences), and anti-CD48 (HM48-1)-APC-Cy7 and anti-CD150/SLAM (TC15-12F12.2)-Pacific blue Abs (BioLegend). Biotinylated primary Abs were visualized with Streptavidin-PE-Cy7 (BD Biosciences). The frequency of GFP (Smad3)^+^ LT-CML stem cells among total GFP (Smad3)^+^ CML-KLS^+^ cells in each recipient was determined using a FACS Aria III cell sorter (BD Biosciences).

### Duolink *in situ* proximity ligation assay

To examine phosphorylation and protein interactions, we used the Duolink *in situ* PLA system (D-PLA; Olink Bioscience), which employs a set of two secondary Abs in which one is conjugated to a minus strand PLA probe and the other is conjugated to a plus strand PLA probe. To visualize the phosphorylation of Smad2, Smad3, p38MAPK, AMPK, Raptor and S6 ribosomal protein, as well as Foxo3a–Smad2, Foxo3a–Smad3 and Foxo3a–phospho-Smad3–Ser208 interactions, LT-CML stem cells, ST-CML stem cells, CD48^+^, MPP and KLS^−^ CML cells that were freshly isolated from tet-inducible CML-affected mice, and LT-normal HSCs that were freshly isolated from healthy control littermates, were immediately fixed with 4% paraformaldehyde for 30 min. For *in vitro* inhibitor experiments, LT-CML stem cells were incubated in 3% O_2_ at 37 °C for 30 min with the appropriate vehicle (control), 5 μM Ly364947 (TGF-β type I receptor kinase Alk5 inhibitor; Merck), 5 μM SB203580 (p38MAPK inhibitor; LC Laboratories), 5 μM GlySar (non-metabolizable dipeptide transporter substrate; Sigma-Aldrich), 5 μM cefadroxil (antibiotic dipeptide transporter competitor; Sigma-Aldrich), 100 nM rapamycin (mTORC1 inhibitor; Cell Signaling Technologies), 10 mM metformin (AMPK activator; TOCRIS Bioscience) or 300 nM flavopiridol (CDK9 inhibitor; Sigma-Aldrich). Treated cells were fixed with 4% paraformaldehyde for 30 min.

In all cases, fixed cells were permeabilized with 0.25% Triton X-100 for 15 min, washed and blocked by incubation in 5% FBS in TBS for 1 h. Blocked cells were incubated overnight at 4 °C with the combinations of Abs indicated in Supplementary Table 1. Primary mouse antibody was used at a 1/25 dilution, primary rabbit antibody at 1/50 and primary goat antibody at 1/50. The proximate binding of these Abs was then detected using D-PLA secondary Abs. Stained slides were mounted using Duolink *in situ* mounting medium with 4′,6-diamidino-2-phenylindole (DAPI) (Sigma-Aldrich), and fluorescent images were acquired by confocal microscopy (FV10i, Olympus) and Photoshop software (Adobe). The number of fluorescent foci per single cell was quantified using Duolink Image Tool software (Olink Bioscience).

As positive and negative controls for Smad3 phosphorylation, we treated LT-CML stem cells *in vitro* for 30 min in 3% O_2_ with TGF-β1 (1 ng ml^−1^; R&D Systems) or Ly364947 (5 μM; Merck), respectively. As a negative control for mTORC1 activation, LT-CML stem cells were treated *in vitro* under the above conditions with rapamycin (100 nM; Cell Signaling Technologies). As a positive control for AMPK activation, LT-CML stem cells were treated *in vitro* under the above conditions with Metformin (10 mM; Cell Signaling Technologies). As indicated in Fig. 4a,d and Supplementary Figs 6–8, the appropriate fluorescent foci were detected (or not) in these control experiments. As a technical negative control for D-PLA, we treated LT-CML stem cells with a single anti-mouse primary Ab and confirmed that no fluorescent foci could be detected, as indicated in Fig. 3a–d. As a technical negative control for interaction between Foxo3a and phospho-Smad3–Ser208, we used *Foxo3a*^*−/−*^ LT-CML stem cells and confirmed that no fluorescent foci could be detected, as indicated in Fig. 4h,i.

### Colony-forming assays

LT-CML stem cells or LT-normal HSCs (1 × 10^3^) were co-cultured on OP-9 stromal cells in 3% O_2_ at 37 °C for 5 days^15^ in the presence of either vehicle (control), the dipeptide transporter competitor cefadroxil (5 μM), or the p38MAPK inhibitors Ly2228820 (5 μM; Santacruz), VX-702 (5 μM; Santacruz) or BIRB796 (5 μM; Calbiochem). Cells were harvested, washed in PBS and plated in semi-solid methylcellulose medium containing SCF, interleukin (IL)-3, IL-6 and erythropoietin (Methocult GF M3434; Stem Cell Technologies). After growth for 7 days under a hypoxic (3% O_2_) condition at 37 °C, colony numbers were counted under a light microscope.

For combination treatments of dipeptide transporter competitor or p38MAPK inhibitor plus TKI, LT-CML stem cells (3 × 10^3^) were first plated on OP-9 stromal cells in the presence of cefadroxil (5 μM), Ly2228820 (5 μM), VX-702 (5 μM) or BIRB796 (5 μM). After 24 h in culture, the cells received additional dimethyl sulfoxide or IM (1 μM; Axon Medchem) and were incubated for another 4 days (total 5 days). Treated cells were washed in PBS, transferred to a semi-solid medium and colony formation after 7 days was assessed as described above.

### Short hairpin RNA targetting *Slc15A2* mRNA

Third-generation HuSH shRNA lentiviral vectors based on pGFP-C-shLenti and carrying 29-mer shRNA sequences targetting the mouse *Slc15A2* gene (TL503323B; mouse *Slc15A2* shRNA B: 5′-GAACCGTTCTGAGGACATTCCAAAGCGAC-3′, and TL503323D; mouse *Slc15A2* shRNA D: 5′-TATCGGCTGATCTCCAAGTGCGGAGTTAA-3′), as well as control scrambled shRNA, were purchased from Origene (Rockville, MD). pCMV-VSV-G and pCMV-dR8.2 dvpr were provided by Addgene (Cambridge, MA). 293TN producer cells (purchased from System Biosciences; Mountain View, CA) were transiently transfected with pGFP-C-shLenti vector (6 μg per 100-mm plate), pCMV-VSV-G (1.5 μg) and pCMV-dR8.2 dvpr (4.5 μg) using FuGene6 (Roche) as described above for retroviral transduction of KLS^+^ cells. At 2 days post transfection, culture supernatants were passed through a 0.45-μm filter and centrifuged at 6,500*g* for 16 h. The virus-containing pellets were resuspended in SF-03 stem cell medium to yield lentiviral solutions carrying shRNA targetting mouse *Slc15A2* mRNA (shRNA B and shRNA D) or scrambled shRNA. CML-KLS^+^ cells and CML-KLS^−^ cells isolated from tet-inducible CML-affected mice were infected with these lentiviruses using Combimag (OZ Biosciences) on a magnetic plate (OZ Biosciences) as described above, and GFP^+^CML-KLS^+^ and GFP^+^CML-KLS^−^ cells were isolated by cell sorting at 3 days post infection. To examine colony-forming ability *in vitro*, these cells were co-cultured on OP-9 stromal cells under hypoxic conditions (3% O_2_) for 5 days, and colony formation was assessed in semin-solid medium as described above.

### Competitive reconstitution assay for normal HSCs

C57BL/6 (CD45.2 for the Ly5 locus) and congenic C57BL/6 (CD45.1 for the Ly5 locus; B6-Ly5.1) mice were purchased from Sankyo-Lab Service. Lethally irradiated (9 Gy) 6-week-old C57BL/6 (CD45.2) recipient mice (female) were reconstituted with 1 × 10^4^ normal KLS^+^ cells (HSCs) from 6-week-old congenic C57BL/6 (CD45.1) (B6-Ly5.1) mice (female) in competition with 5 × 10^5^ unfractionated BM MNCs from 6-week-old C57BL/6 (CD45.2) mice (female). Transplanted recipients then received vehicle or cefadroxil (36 mg kg^−1^ per day) from day 0 to 12 weeks post transplantation. Reconstitution of donor-derived cells (CD45.1) was monitored at 4, 8 and 12 weeks post transplantation by flow cytometric analysis of peripheral blood mononuclear cells stained with monoclonal antibodies (mAbs) against CD45.2 (104)-FITC (eBiosciences) and CD45.1(A20)-PE (BD Biosciences).

### Serial transplantation of CML stem cells

To evaluate the CML stem cell activity after treatment of mice with cefadroxil and/or IM *in vivo*, the frequency of GFP/BCR-ABL1^+^CML-KLS^+^ cells in treated BCR-ABL1-CML-affected mice was determined. To evaluate the retention of the disease-initiating capacity by CML stem cells after treatment of mice with cefadroxil *in vivo*, subsequent secondary transplantation of the GFP/BCR-ABL1^+^CML-KLS^+^ cells was performed. Briefly, BCR-ABL1-CML-affected mice received IM (100 mg kg^−1^ per day) and/or cefadroxil (36 mg kg^−1^ per day) by oral gavage for 30 days post BM transplantation as described above. The frequency of GFP/BCR-ABL1^+^CML-KLS^+^ cells among total GFP/BCR-ABL1^+^CML cells isolated from BM MNCs acquired from the two hindlimbs of treated CML-affected mice was assessed by flow cytometry. Freshly purified GFP/BCR-ABL1^+^CML-KLS^+^ cells (3 × 10^4^) were then serially transplanted into a second set of lethally irradiated (9 Gy) 6-week-old congenic recipient mice (female) along with 5 × 10^5^ normal BM MNCs from 6-week-old C57BL/6 mice (female). Mouse survival and disease recurrence were monitored for up to 90 days.

### *SLC15A2* mRNA expression in human CML patients

Data on *SLC15A2* mRNA levels in human CML patients were obtained from a public database gene expression omnibus (GEO, ID: GSE33075)^54^ that contains microarray analyses of nine healthy donors, nine CML patients before treatment and the same nine CML patients 1 month after treatment with IM^55^. A one-tailed *t-*test was used to compare *SLC15A2* expression between CML patients before and after IM treatment. The one-tailed *t*-test for independent samples was used to compare *SLC15A2* expression between CML patients and healthy donors.

### Colony-forming capacity of human CML-LICs

Viable BM MNCs from three human patients with chronic phase CML were purchased from Allcells (#06–255, #06–620 and #147742, Alameda, CA, USA). We stained these cells with anti-CD34(8G12), anti-CD38(HIT2), anti-CD3(SK7), anti-CD16(3G8), anti-CD19(SJ25C1), anti-CD20(L27), anti-CD14(MφP9) and anti-CD56(NCAM16.2) Abs (BD Biosciences). A mixture of mAbs recognizing CD3, CD16, CD19, CD20, CD14 and CD56 was used to identify Lin^−^ cells, and CD34^+^CD38^−^Lin^−^ cells were purified^15^. To determine the effects of treatment with cefadroxil (5 μM) alone, or with a combination of cefadroxil plus IM (1 μM; Axon Medchem) or dasatinib (500 nM; LC laboratories), CD34^+^CD38^−^Lin^−^ cells were cultured on OP-9 stromal cells under hypoxic (3% O_2_) conditions. To determine the effects of treatment with Ly2228820 (5 μM) alone, or with a combination of Ly2228820 plus dasatinib (500 nM; LC laboratories), CD34^+^CD38^−^Lin^−^ cells were cultured on OP-9 stromal cells under hypoxic (3% O_2_) conditions. After harvesting and washing in PBS, the colony-forming ability of primitive human CML-LICs was evaluated by culture in semi-solid methylcellulose medium containing SCF, granulocyte–macrophage colony-stimulating factor (CSF), IL-3, IL-6, granulocyte CSF and erythropoietin (Methocult GF^+^ H4435; Stem Cell Technologies). After growth for 7 days at 37 °C under hypoxic (3% O_2_) conditions, colony numbers were counted under a light microscope.

### Statistical analyses

Statistical differences were determined using the unpaired Student's *t*-test, one-tailed *t*-test and Welch's *t*-test for *P* values, and a log-rank non-parametric test for survival curves.

## Additional information

**How to cite this article:** Naka, K. *et al.* Dipeptide species regulate p38MAPK–Smad3 signalling to maintain chronic myelogenous leukaemia stem cells. *Nat. Commun.* 6:8039 doi: 10.1038/ncomms9039 (2015).

## Supplementary Material

Supplementary Figures and Table Supplementary Figures 1-12 and Supplementary Table 1

Supplementary Data 1 Metabolomics data for CML stem cells and normal HSCs

Supplementary Data 2 Next-generation RNA-sequencing results for LT-CML stem cells and normal LT-HSCs
