# Supplementary material for: Dipeptide species regulate p38MAPK–Smad3 signalling to maintain chronic myelogenous leukaemia stem cells
Source: Nat Commun. 2015 Aug 20;6:8039. doi: 10.1038/ncomms9039 (PMC4560789; doi:10.1038/ncomms9039)
Supplement: Supplementary Figures and Table — Supplementary Figures 1-12 and Supplementary Table 1 [file ncomms9039-s1.pdf]

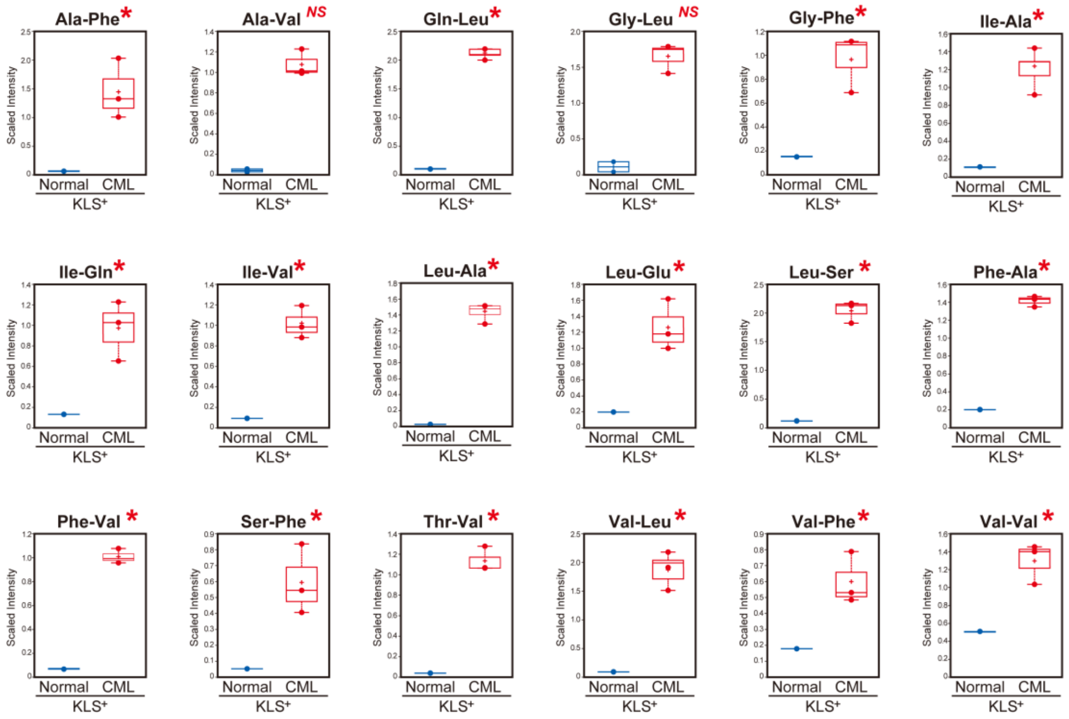

Supplementary Figure 1. Accumulation of various dipeptide species in CML stem cells.

Levels of the indicated dipeptides were determined using the metabolomics approach described in **Methods** and in **Fig.1** for KLS$^+$ cells isolated from tet-inducible CML-affected *Tal1-tTA*$^+$ *TRE-BCR-ABL1*$^+$ mice (n=4 mice in each of 3 experiments) and normal littermate *Tal1-tTA*$^+$ mice (n=6 mice in each of 2 experiments) at 5 wks after DOX withdrawal. Amounts of dipeptides in normal KLS$^+$ and CML-KLS$^+$ cells were plotted in whisker boxes. Cross, mean value; horizontal line across the box, median value; error bars, maximum and minimum of distribution; dot, extreme data point (*, $P<0.05$; Normal-KLS$^+$ vs. CML-KLS$^+$; Welch's *t*-test; *NS*, Not significant). See also **Fig.1a-c**. For quantitation summary, see **Fig.1d**.

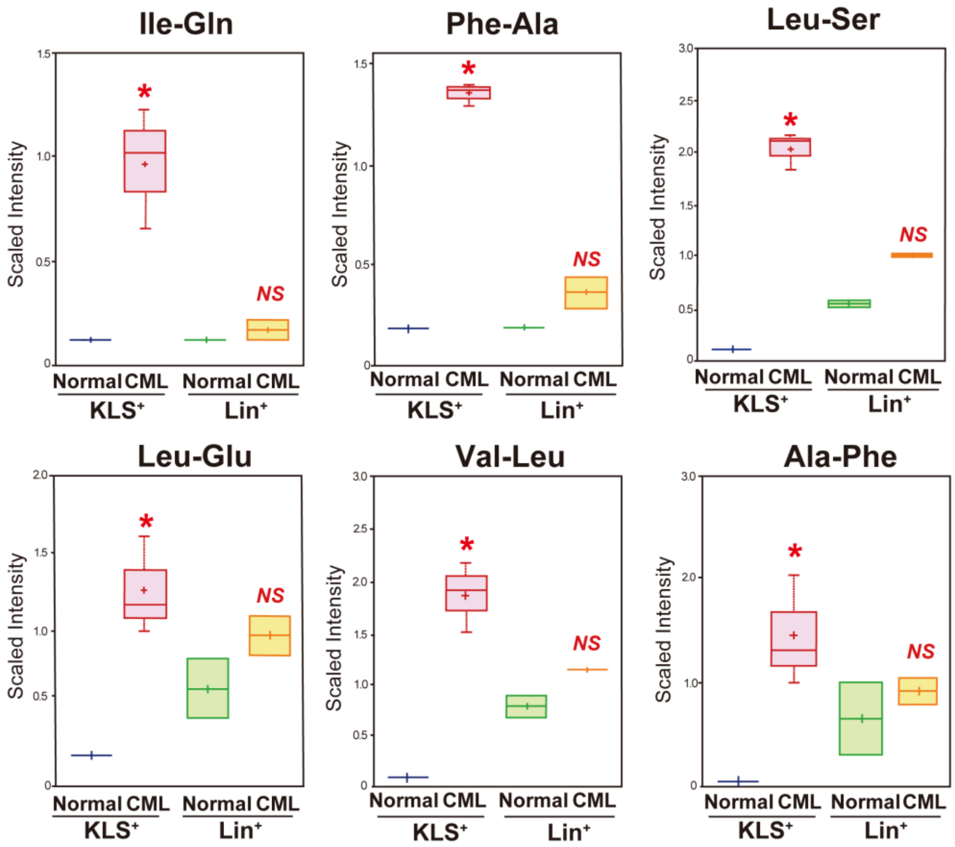

Supplementary Figure 2. Comparison of dipeptide species between CML stem cells and differentiated CML cells.

Levels of the indicated dipeptides were determined using the metabolomics approach described in **Methods** for immature KLS^+ cells and differentiated Lin^+ cells isolated from tet-inducible CML-affected *Tal1-tTA*$^+$ *TRE-BCR-ABL1*$^+$ mice (n=4 mice in each of 3 experiments) and normal littermate *Tal1-tTA*$^+$ mice (n=6 mice in each of 2 experiments) at 5 wks after DOX withdrawal. Results are the scaled intensity values for a given dipeptide in CML-KLS^+, CML-Lin^+, normal KLS^+, and normal Lin^+ cells. Amounts of dipeptides were plotted in whisker boxes. Cross, mean value; horizontal line across the box, median value; error bars, maximum and minimum of distribution (*, $P<0.05$; Normal vs. CML; Welch's *t*-test; *NS*, Not significant). See also **Fig.1c,d**. and **Supplementary Fig.1**.

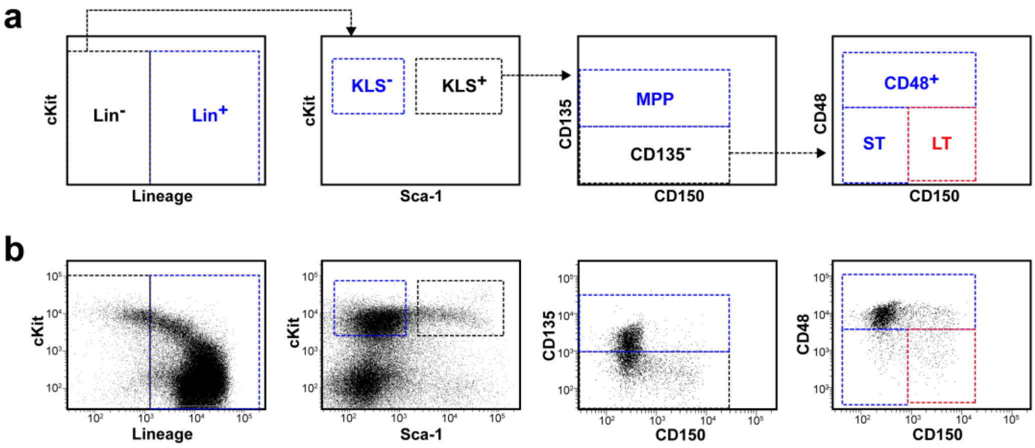

Supplementary Figure 3. Fractionation of subpopulations of CML cells.

Total bone marrow (BM) mononuclear cells (MNCs) were isolated from the two hind limbs of tet-inducible CML-affected mice. These cells were immunostained to detect lineage (Lin) markers (CD4, CD8, B220, Mac1, Gr-1 and Ter119), as well as Sca-1, cKit, CD135, CD48 and CD150, and analysed by flow cytometry. Cells were classified as: Lin$^+$ CML cells (Lin$^+$); Lin$^-$ CML cells (Lin$^-$); KLS$^-$ CML cells (cKit$^+$Lin$^-$Sca-1$^-$) (KLS$^-$); KLS$^+$ CML cells (cKit$^+$Lin$^-$Sca-1$^+$) (KLS$^+$); multipotent progenitor (MPP)-like CML cells (CD135$^+$KLS$^+$) (MPP); CD48$^+$ CML cells (CD48$^+$CD135$^-$KLS$^+$) (CD48$^+$); ST-CML stem cells (CD150$^-$CD48$^-$CD135$^-$KLS$^+$) (ST); or the most primitive LT-CML stem cells (CD150$^+$CD48$^-$CD135$^-$KLS$^+$) (LT). The gating pattern for cell sorting (**a**) and representative flow cytometric data (**b**) are shown.

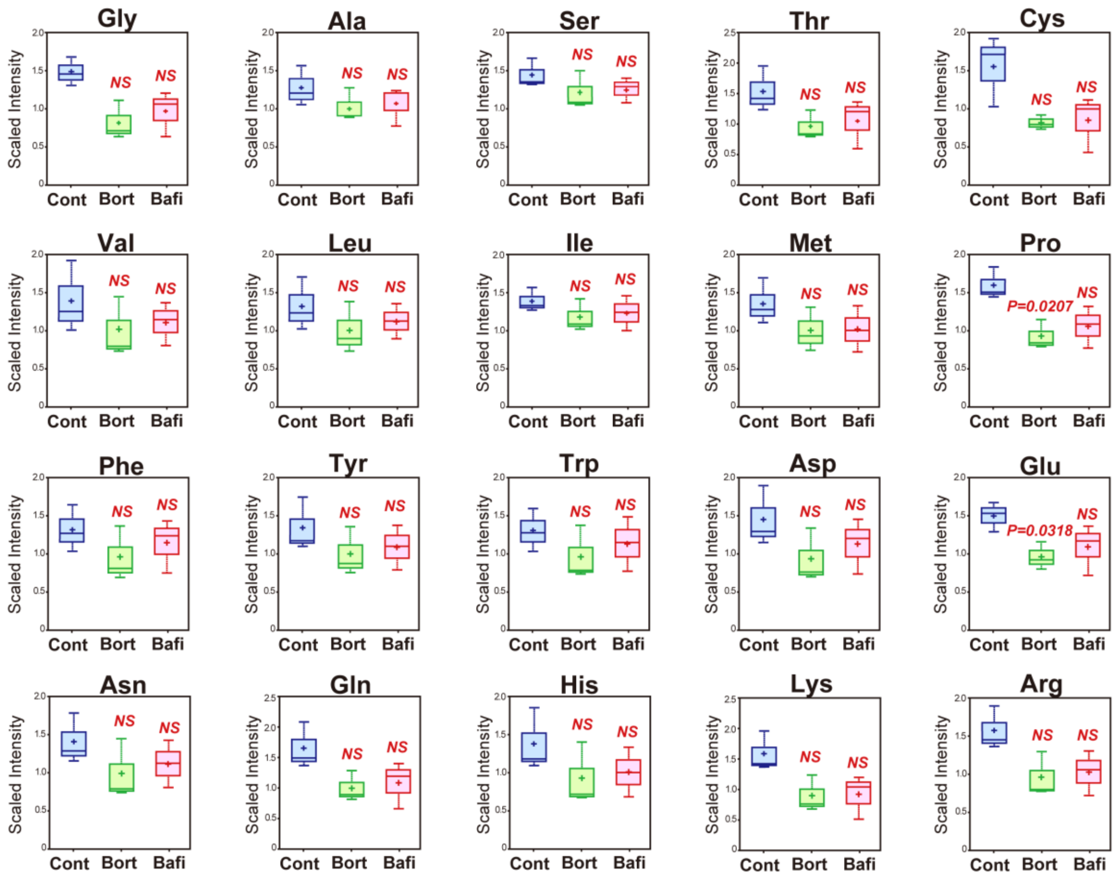

Supplementary Figure 4. Effect of proteasome and autophagy inhibitors on amino acid levels in CML stem cells.

Metabolomics analysis of the indicated individual amino acids in CML-KLS^{+} cells treated *in vitro* for 2hr with vehicle (DMSO; control; Cont), Bortezomib (100nM) or Bafilomycin A1 (100nM). Data are scaled intensity values analysed as in **Fig.1b** from 3 independent experiments. Amounts of amino acids were plotted in whisker boxes. Cross, mean value; horizontal line across the box, median value; error bars, maximum and minimum of distribution (*, $P<0.05$ compared with control; Welch's *t*-test; *NS*, Not significant). See also **Supplementary Fig.5**.

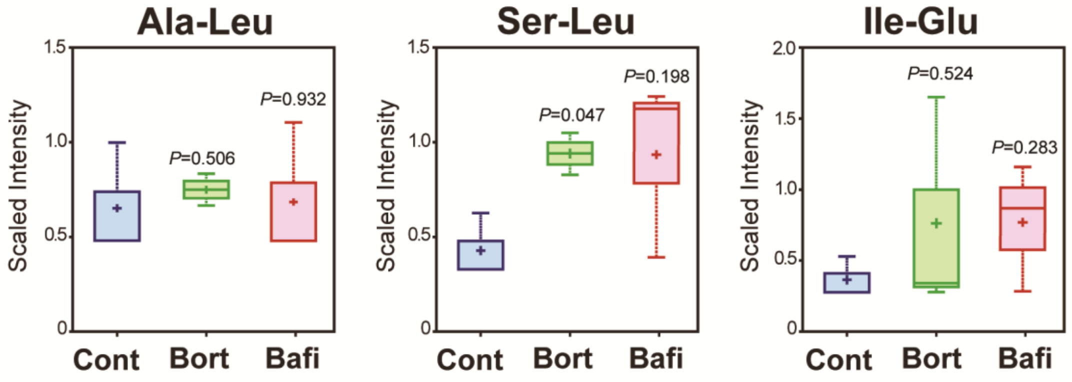

Supplementary Figure 5. Effect of proteasome and autophagy inhibitors on dipeptide levels in CML stem cells.

Metabolomics analysis of the indicated dipeptide species in CML-KLS$^+$ cells treated *in vitro* for 2h with vehicle (DMSO; control; Cont), Bortezomib (100nM; Bort) or Bafilomycin A1 (100nM; Bafi). Data are scaled intensity values obtained from 3 independent experiments and analysed as in **Fig.1c**. Amounts of dipeptides were plotted in whisker boxes. Cross, mean value; horizontal line across the box, median value; error bars, maximum and minimum of distribution. *P*-value compared with control was measured by Welch's *t*-test. See also **Supplementary Fig.4**.

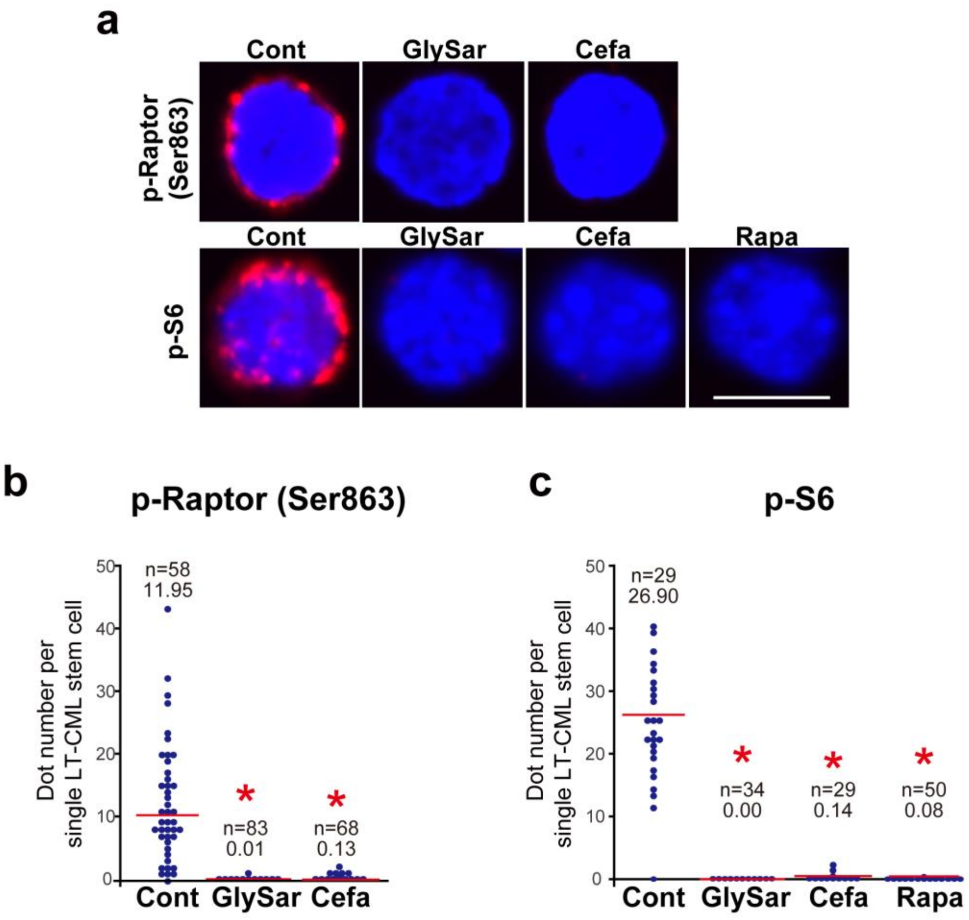

Supplementary Figure 6. Inhibition of Slc15A2-mediated dipeptide uptake attenuates mTORC1-mediated nutrient signalling in LT-CML stem cells.

(a) Duolink *in situ* PLA (D-PLA) imaging of the phosphorylation of Raptor-Ser863 and S6 in LT-CML stem cells that were treated for 30 min with vehicle (Control), 5μM GlySar, or 5μM Cefadroxil under hypoxic (3% O_2) conditions. Rapamycin (Rapa; 100nM) is a positive control for the inhibition of mTORC1 signalling. Nuclei were visualised using DAPI. The combinations of primary antibodies (Abs) used are listed in **Supplementary Table 1**. Scale bar, 10μm. Results are representative of 3 trials.

(**b,c**) Quantitation of dot number of phospho-Raptor-Ser863 (**b**) and phospho-S6 (**c**) per single LT-CML stem cell determined from the 3 experiments in (**a)** using Duolink Image Tool software. The mean dot number (red line) appears under total cells number (n) (*****, P<0.00005 compared with control; Student's *t*-test).

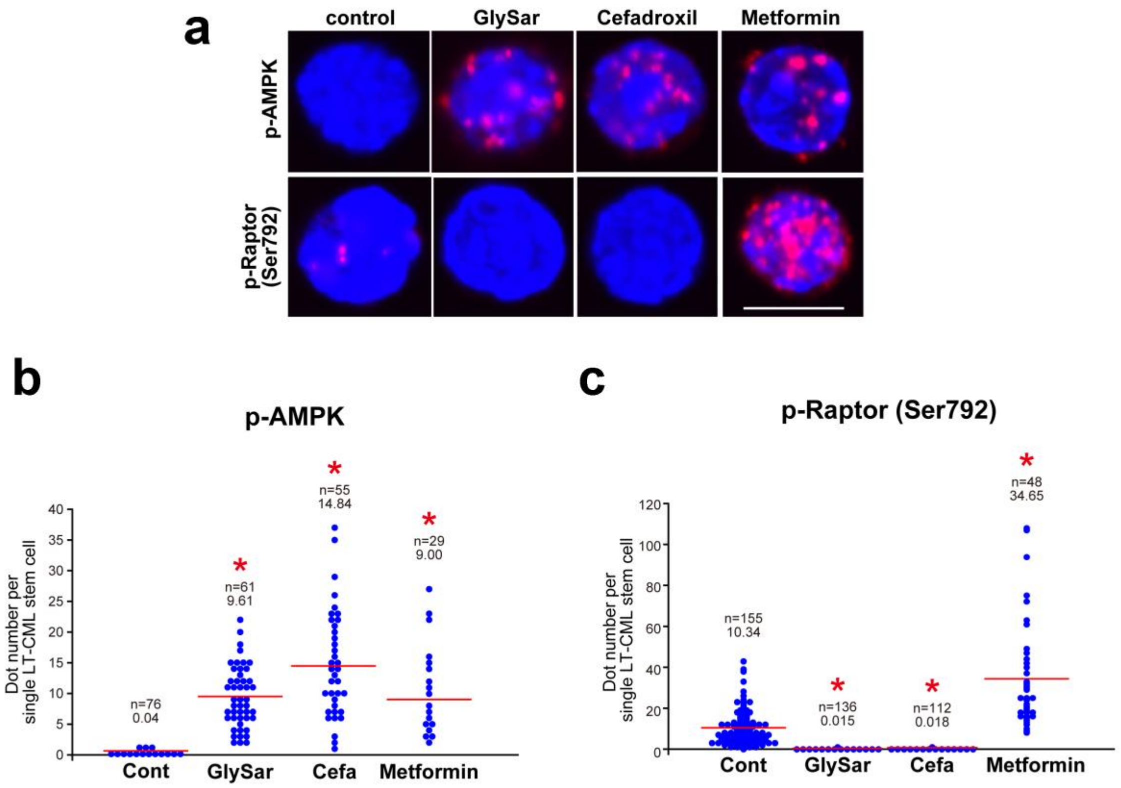

Supplementary Figure 7. AMPK does not inhibit mTORC1 pathway in CML stem cells after treatment with Slc15A2 dipeptide transporter inhibitors.

(**a**) Duolink *in situ* PLA (D-PLA) imaging of (top) AMPK phosphorylation at Thr172, and (bottom) Raptor phosphorylation at Ser792, in freshly isolated LT-CML stem cells that were treated for 30 min with vehicle (Control), 5μM GlySar, or 5μM Cefadroxil under hypoxic (3% O_2) conditions. The combinations of primary antibodies (Abs) used are listed in **Supplementary Table 1**. Treatment with Metformin (AMPK activator; 10 mM) was a technical positive control for AMPK phosphorylation and for Raptor-Ser792 phosphorylation. Nuclei were visualised using DAPI. Scale bar, 10μm. Results are representative of 3 trials.

(**b,c**) Quantitation of dot numbers of phospho-AMPK (**b**) and phospho-Raptor-Ser792 (**c**) per single LT-CML stem cell determined from the 3 experiments in (**a**) using Duolink Image Tool software. The mean dot number (red line) appears under total cells number (n) (*****, $P<0.00005$ compared with control; Student's *t*-test).

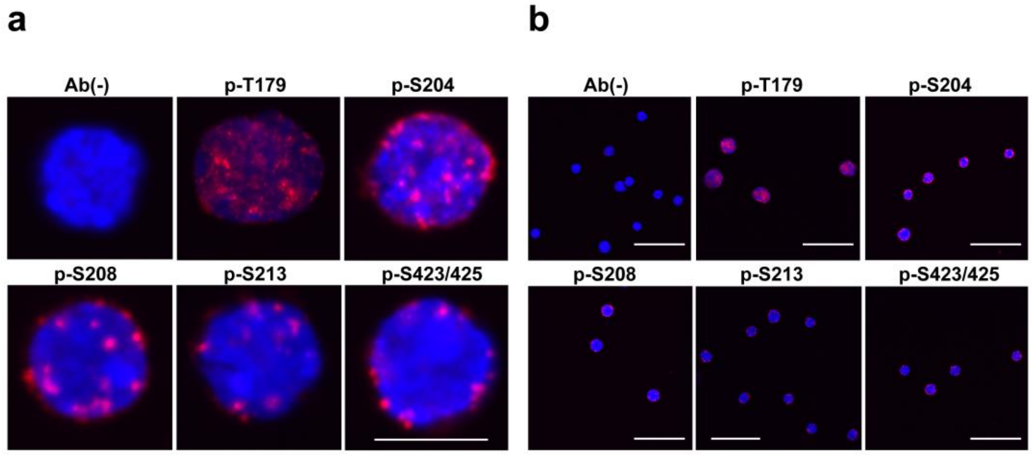

Supplementary Figure 8. Site-specific Smad3 phosphorylation in LT-CML cells induced by TGF-β1.

D-PLA imaging of freshly isolated LT-CML stem cells that were treated with 1ng ml^{-1} TGF-β1 for 30 min as a technical positive control for enforced Smad3 phosphorylation at Thr179, Ser204, Ser208, Ser213 and Ser423/Ser425. (**a**) High magnification images. Scale bar, 10μm. (**b**) Low magnification images. Scale bars, 50μm. The combination of primary antibodies used is listed in **Supplementary Table 1**. Ab (-), technical negative control using a single mouse anti-Smad3 antibody. Nuclei were visualised using DAPI (blue). See also **Fig.3c,d**.

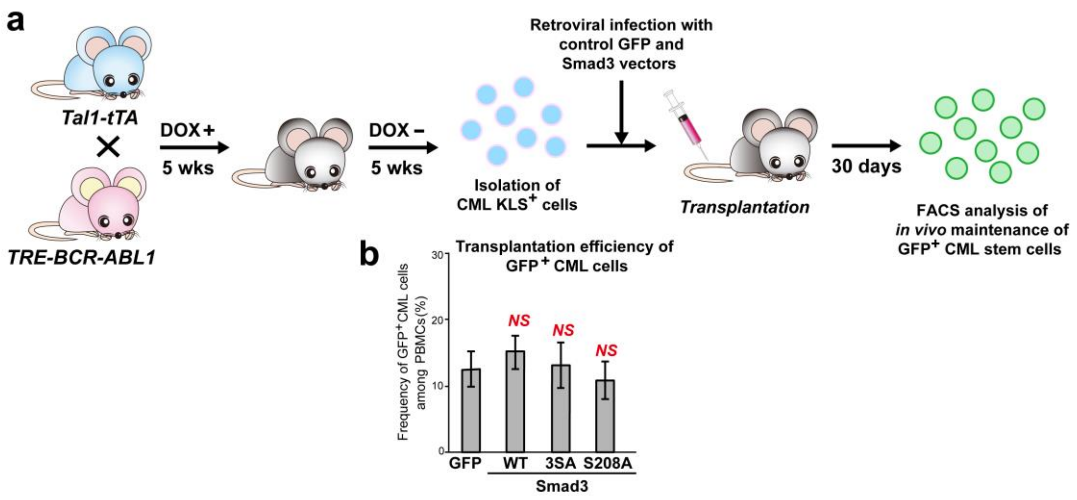

Supplementary Figure 9. Transplantation of retrovirally-transduced Smad3-expressing KLS$^+$ cells to evaluate LT-CML stem cell maintenance *in vivo*.
(**a**) Schematic illustration of the experimental protocol. Tet-inducible CML-affected mice were generated by crossing the indicated strains. Doxycycline (DOX) was supplied to the progeny for 5 wks and then withdrawn for 5 wks to induce CML-like disease. CML KLS$^+$ cells were purified from BM MNCs isolated from the two hind limbs of CML-affected mice and cultured overnight before incubation with solutions of retroviral vectors expressing control GFP, human Smad3-WT, Smad3-3SA or Smad3-S208A. Retrovirally-infected CML KLS$^+$ cells were transplanted into irradiated congenic recipient mice (1.0-1.5x10^5 cells/mouse). After 30 days, BM MNCs were analysed by flow cytometry to detect GFP$^+$ CML stem cells.
(**b**) Quantitation of transplantation efficiency of CML KLS$^+$ cells infected with control-GFP or the indicated Smad3-expressing retroviral vectors. The frequency of GFP$^+$ CML cells among total peripheral blood mononuclear cells (PBMCs) was assessed at 4 wks post-transplantation by flow cytometry. Results are the mean ± s.d. (female; n=3 mice per group) and are representative of 3 independent trials (P-value compared with GFP$^+$ CML cells; Student's t-test). See also **Fig.3h-j**.

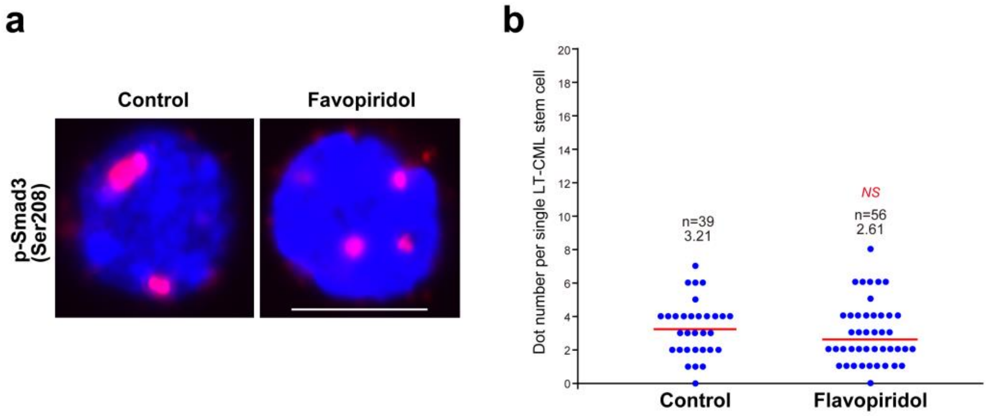

Supplementary Figure 10. The cyclin-dependent kinase 9 (CDK9) inhibitor Flavopiridol does not affect Smad3-Ser208 phosphorylation in LT-CML stem cells. **(a)** D-PLA imaging of Smad3-Ser208 phosphorylation in freshly isolated LT-CML stem cells that were treated for 30 min with vehicle (Control) or 300nM Flavopiridol (CDK9 inhibitor) under hypoxic (3% O_2) conditions. The combination of primary antibodies used is listed in **Supplementary Table 1**. Nuclei were visualised using DAPI. Scale bar, 10μm.

(**b**) Quantitation of dot numbers of phospho-Smad3-Ser208 per single LT-CML stem cell determined from the 3 experiments in (**a**) using Duolink Image Tool software. The mean dot number (red line) appears under total cells number (n). The statistical significance among control vs. Flavopiridol was measured by Student's *t*-test. *NS*, not significant.

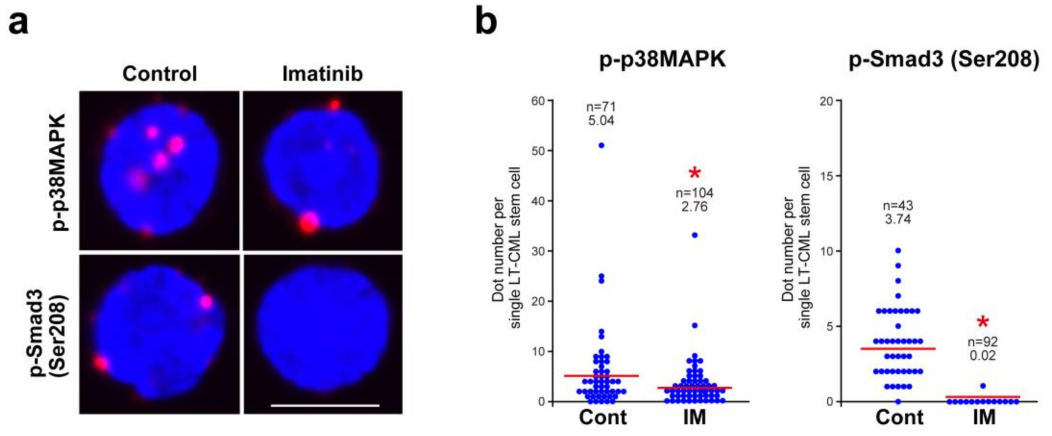

Supplementary Figure 11. Attenuated phosphorylation of p38MAPK and Smad3-Ser208 in LT-CML stem cells after treatment with Imatinib.

(a) D-PLA imaging of p38MAPK and Smad3-Ser208 phosphorylation in freshly isolated LT-CML stem cells that were treated for 30 min with vehicle (Control) or 1μM imatinib (IM) under hypoxic (3% O_2) conditions. The combination of primary antibodies used is listed in **Supplementary Table 1**. Nuclei were visualised using DAPI. Scale bar, 10μm.

(**b**) Quantitation of dot numbers of phospho-p38MAPK and phospho-Smad3-Ser208 per single LT-CML stem cell determined from the 3 experiments in (**a**) using Duolink Image Tool software (*****, P<0.00005 compared with control; Student's *t*-test).

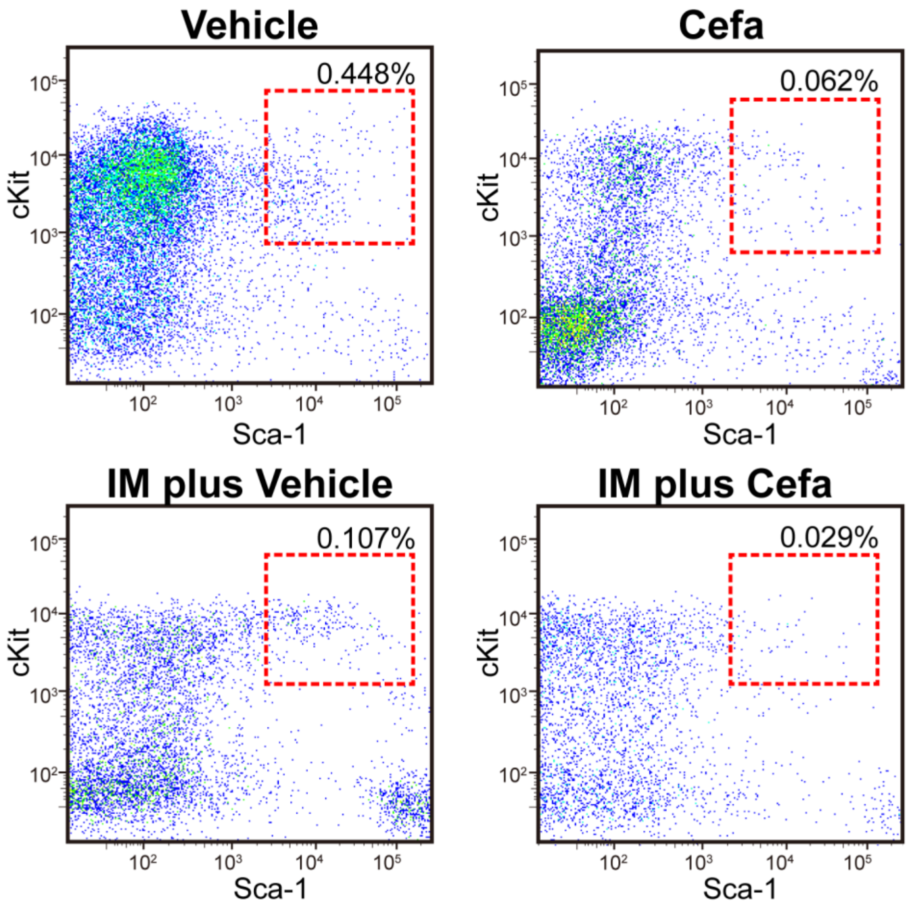

Supplementary Figure 12. Cefadroxil administration *in vivo* reduces the frequency of GFP/BCR-ABL1^{+}CML-KLS^{+} cells in mice.

Lethally irradiated C57BL/6 recipient mice (female) were transplanted with HSCs transduced with the *GFP/BCR-ABL1* oncogene. At 8 days post-transplantation, CML-affected mice received: vehicle alone (female; *n*=3), vehicle plus Cefadroxil (Cefa; 36mg Kg^{-1}day^{-1}; female; *n*=4), vehicle plus IM (100mg Kg^{-1}day^{-1}; female; *n*=4), or IM plus Cefadroxil (female; *n*=4). Treatment was delivered by oral gavage for 30 days post-BM transplantation. Data shown are representative flow cytometric analyses highlighting GFP/BCR-ABL1^{+}CML KLS^{+} cells (red rectangles). Cells were gated on GFP^{+}, Lineage^{-}. See also **Fig.6e,f**.

Supplementary Table 1. Antibody combinations for Duolink *in situ* PLA analyses

Purpose	Detection	PLA plus strand	PLA minus strand
Phosphorylation	p-Ser465/467 Smad2	Goat anti-phospho-Ser465/467 Smad2/ phospho-Ser423/425 Smad3 (Santacruz, Sc-11769)	Rabbit anti-Smad2 (D43B4) (Cell Signaling, #5339)
	p-Thr179 Smad3	Rabbit anti-Smad3 (C67H9) (Cell Signaling, #9523)	Mouse anti-phospho-Thr179 Smad3/ phospho-Thr220 Smad2 (IBL, 1A1)
	p-Ser204 Smad3	Rabbit anti-phospho-Ser204 Smad3 (Abcam, ab63402)	Mouse anti-Smad3 (Abcam, ab75512)
	p-Ser208 Smad3	Rabbit anti-phospho-Ser208 Smad3 (Abcam, ab138659)	Mouse anti-Smad3 (Abcam, ab75512)
	p-Ser213 Smad3	Rabbit anti-phospho-Ser213 Smad3 (Millipore, ABS48)	Mouse anti-Smad3 (Abcam, ab75512)
	p-Ser423/425 Smad3	Rabbit anti-phospho-Ser423/425 Smad3 (Abcam, ab51451)	Mouse anti-Smad3 (Abcam, ab75512)
	p-Thr180/Tyr182 p38MAPK	Rabbit anti-p38MAPK (Cell Signaling, D13E1 #8690)	Mouse anti-phospho-Thr180/Tyr182 p38MAPK (Cell Signaling, 28D10 #9216)
	p-Thr172 AMPK	Rabbit anti-phospho-Thr172 AMPK (40H9) (Cell Signaling, #2535)	Mouse anti-AMPKa (F6) (Cell Signaling, #2793)
	p-Ser792 Raptor	Rabbit anti-phospho-Ser792 Raptor (Cell Signaling, #2083)	Mouse anti-Raptor (10E10) (Santacruz, Sc-81537)
	p-Ser863 Raptor	Rabbit anti-phospho-Ser863 Raptor (Santacruz, Sc-130214)	Mouse anti-Raptor (10E10) (Santacruz, Sc-81537)
	p-Ser235/236 S6 ribosomal protein	Rabbit anti-phospho-Ser235/236 S6 ribosomal protein (D57.2.2E) (Cell Signaling, #4858)	Mouse anti-S6 ribosomal protein (54D2) (Cell Signaling, #2317)
Interaction	Smad2-Foxo3a	Rabbit anti-Smad2 (D43B4) (Cell Signaling, #5339)	Mouse anti-FKHRL1(FR1) (Sigma, F1304)
	Foxo3a-Smad3	Rabbit anti-Foxo3a (75D8) (Cell Signaling, #2497)	Mouse anti-Smad3 (Abcam, ab75512)
	p-Ser208 Smad3-Foxo3a	Rabbit anti-phospho-Ser208 Smad3 (Abcam, ab138659)	Goat anti-Foxo3a (Abcam, ab17026)
